# Staining Tissues with Basic Blue 7: A New Dual-Polarity Matrix for MALDI Mass Spectrometry Imaging

**DOI:** 10.1021/acs.analchem.4c05244

**Published:** 2025-01-30

**Authors:** Michal Javorek, Michal Hendrych, Kateřina Ondráková, Jan Preisler, Antonín Bednařík

**Affiliations:** † Department of Chemistry, Faculty of Science, Masaryk University, Brno 625 00, Czech Republic; ‡ First Department of Pathology, St. Anne’s University Hospital, Brno 602 00, Czech Republic; § First Department of Pathology, Faculty of Medicine, Masaryk University, Brno 625 00, Czech Republic

## Abstract

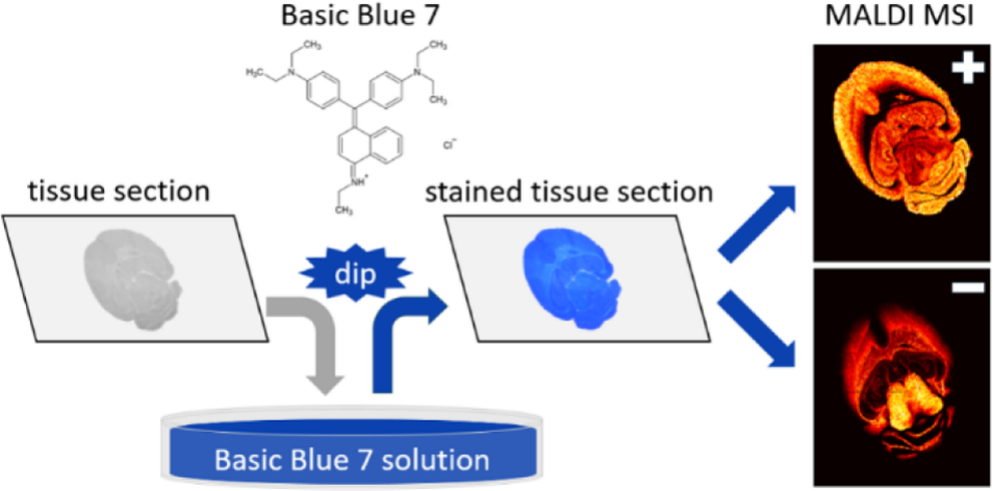

Obtaining high-quality matrix-assisted laser desorption/ionization mass spectrometry (MALDI MS) images and the reproducibility of the technique depend strongly on the sample preparation protocol. The most crucial part is the application of the MALDI matrix, which often relies on expensive spraying or sublimation coaters. In this work, we present a new dual-polarity matrix for MALDI mass spectrometry imaging (MSI): Basic Blue 7 (BB7), which belongs to the group of triarylmethane dyes. Thanks to its good solubility in water, this matrix allows a quick and simple sample preparation protocol without the need for sophisticated spraying or sublimation instrumentation: dipping the glass with tissue into the dye solution. This technique closely resembles the staining methods employed in classical histopathology. The technique is demonstrated on MSI of lipids in mouse brain sections in positive and negative ion modes using a subatmospheric pressure MALDI source coupled with an orbital trap mass spectrometer. The results are compared with traditional matrices, such as 2,5-dihydroxybenzoic acid (DHB) and 1,5-diaminonaphthalene (DAN). BB7 excels, especially in negative ion mode, offering low background signals and high signal intensities of many lipid classes. Furthermore, the stained tissue can simply be inspected visually and allows basic histopathology annotation prior to MSI. Here, we demonstrate that staining offers excellent image quality, reproducible sample preparation, and the potential for automation and utilization for high spatial resolution MSI.

## Introduction

Matrix-assisted laser desorption/ionization mass spectrometry imaging (MALDI MSI) is a powerful label-free technique for studying the spatial distribution of various biomolecules in biological tissues.^1^ Despite the fast growth of this technique over the past decade, there are still many challenges in MALDI MSI that have not been completely resolved.^2^ For example, complex spectra of commonly used matrices in low mass-to-charge (*m*/*z*) regions restraining the analysis of small molecules;^3^ limited sensitivity for certain molecule classes;^2,4^ reproducible sample preparation protocols^5^ often requiring expensive spraying/sublimation equipment;^6,7^ compatibility with other imaging and histology techniques;^8,9^ and quantification^10^ or increasing spatial resolution to micrometer ranges.^11^ Although a universal MALDI matrix does not exist, many of these individual challenges can be addressed by selecting the proper matrix with suitable properties.^12^

A typical MALDI matrix is a small organic molecule that forms cocrystals with analytes, efficiently absorbs laser irradiation, and ionizes the analytes, primarily by proton transfer.^13^ An ideal matrix produces simple MALDI mass spectra containing no or only a few signals originating from the matrix itself, to avoid interference with analyte signals. Furthermore, it should be able to provide quality spectra of multiple analytes in both positive and negative ion modes, i.e., be a so-called dual-polarity matrix.^14^ Typically, a dual-polarity matrix has a high proton affinity and low deprotonation energy to produce both [M + H]^+^ and [M – H]^−^ ions, which are provided by both acidic and basic functional groups incorporated into its structure. As there is no ideal or universal MALDI matrix for analysis and MSI of different classes of biomolecules (lipids, proteins, etc.),^12^ different matrices are applied, and the quest for searching for novel matrices with better properties is a seemingly never-ending task.^15^ Examples of recently introduced dual-polarity matrices for MALDI MSI are derivates of anthranilic acid,^14^ cyanographene^16^ or (*E*)-4-(2,5-dihydroxyphenyl)but-3-en-2-one.^17^ Interesting classes of MALDI matrices are based on organic dyes, including fluorescent dyes, 2,3-dicyanohydroquinone,^13^ IR-780,^18^ or heterocyclic-based dyes.^19^

Lipids represent a widely studied group of biomolecules having a large diagnostic potential, thanks to the many essential functions they fulfill in an organism, ranging from cell membrane constitution to energy storage and molecular signaling.^20^ With the variety of functions also comes a variety of lipid structures, which implies that not all classes of lipids ionize well in both positive/negative modes or using a single MALDI matrix.^21^ Cholesterol^22^ or phosphatidylcholines (PCs) are routinely analyzed in positive ion mode.^23,24^ The negative mode is more suitable for the analysis of phosphatidylethanolamines (PEs), phosphatidylinositols (PIs), or ceramides.^25^ Common matrices for successful MALDI MSI of lipids are 2,5-dihydroxybenzoic acid (DHB) or sinapinic acid (SA),^26^ useful mostly in positive mode. In negative mode, 9-aminoacridine (9-AA)^27^ or 2-(2-aminoethylamino)-5-nitropyridine^28^ are most useful, and 1,5-diaminonaphthalene (DAN) in both polarities.^29^

Obtaining high-quality MS images and reproducibility of the technique depend on the sample preparation protocol. Perhaps the most crucial part is the application of the MALDI matrix.^30^ With increasing MALDI MSI spatial resolution to the micrometer range, the demands for matrix application also increase in terms of better homogeneity of the matrix layer and smaller size of produced crystals.^31^ In addition, preserving the structural integrity and preventing analyte delocalization during sample preparation is essential.^32^ Currently, two approaches dominate sample preparation for MALDI MSI: pneumatic spraying and sublimation. Spraying can be a relatively fast and simple method to create a homogeneous matrix layer.^33^ Depending on the parameters of the spraying, a wet or dry aerosol is generated. Wet spray can increase the extraction of analytes from tissues and lead to augmented signals. However, an excessive amount of wet matrix solution can lead to the delocalization of analytes.^34^ On the other hand, sublimation is a dry method that efficiently prevents the delocalization of analyzed molecules.^35^ After matrix sublimation, recrystallization is recommended for additional extraction of analytes from the tissue into the matrix layer, increasing signals up to an order of magnitude.^36^ More specialized methods of matrix application, such as dry coatings^37,38^ or incorporation of the matrix in agar medium, have been proposed.^39^ Tissue staining with dyes traditionally used for histological staining of lipids, namely Sudan Black B, Oil Red O, and Nile Blue A, has also been investigated.^19^ However, for successful MSI using these dyes, previous lipid insolubilization with a chromate solution or chemical fixation with osmium tetroxide is necessary.

**Figure 1:**
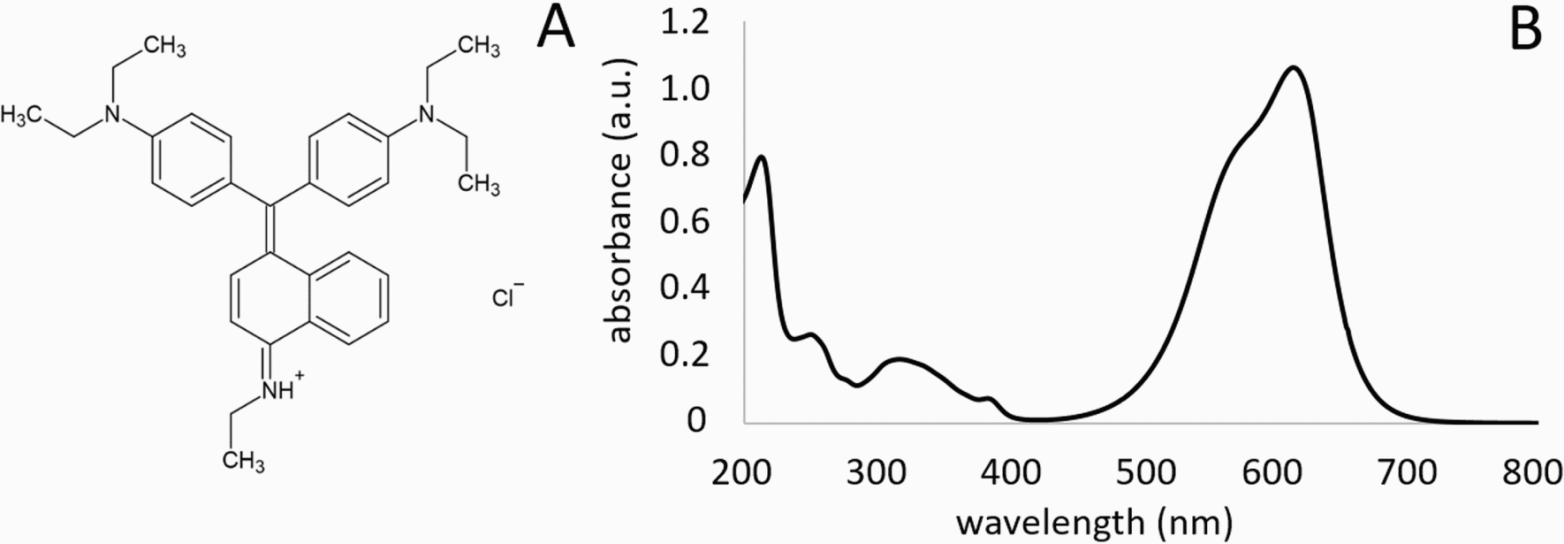
A) Structure and B) UV–vis absorption spectrum of BB7.

In this work, we present a new dual-polarity matrix for MALDI MSI: Basic Blue 7 (BB7), with the systematic name *N*-(4-((4-(diethylamino)phenyl)(4-(ethylamino)naphthalen-1-yl)methylene)cyclohexa-2,5-dien-1-ylidene)-*N*-ethylethanaminium chloride (Figure 1A). BB7 belongs to the group of triarylmethane dyes.^40^ It is often used to dye paper, wool, silk, nylon, or as an ink.^41^ Owing to adequate solubility in water (∼20 mg·mL^–1^), this matrix allows a simple sample preparation protocol resembling the staining methods of classical histopathology without previous chemical fixation of the tissues: dipping the glass with mounted tissue into the dye solution. Tissue staining with BB7 is explored in detail, and its MALDI MSI performance is compared with traditional sample preparation protocols using DHB and DAN matrices.

## Experimental Section

### Chemicals

MALDI matrices 2,5-dihydroxybenzoic acid (DHB), 1,5-diaminonaphthalene (DAN), BB7 analytical standard (≥95%), and BB7 (dye content ≥ 90%) were purchased from Sigma-Aldrich (St. Louis, MO, USA). Ammonium formate (PA grade) was obtained from POCh (Gliwice, Poland). Lipid standards (PC 34:1, PE 34:1, PC 40:0) were acquired from Avanti Polar Lipids, Inc. (Alabaster, AL, USA). Methanol (LC-MS grade) was purchased from Biosolve Chimie (Dieuze, France), and dichloromethane was purchased from Honeywell (Charlotte, NC, USA).

Mouse brains from BALB/c mice were donated by the Institute of Analytical Chemistry of the Czech Academy of Sciences. A pig brain was purchased from a local butcher.

### Dried Droplet Analysis

For dried droplet analysis, a hydrophobic gel pen was used to draw compartments around 3 × 3 mm on the microscope glass, where 1 μL of lipid standard (10 mg·mL^–1^) or pig brain lipid extract in methanol was deposited. After solvent drying, the spot was overlaid with a 10 mg·mL^–1^ BB7 water solution, allowed to dry, and analyzed by MALDI MS. A modified Folch extraction was employed for the preparation of brain lipid extract using a dichloromethane/methanol solution (2:1, V/V, 4 °C), as described in our previous work.^42^

### Preparation of Tissue Sections

Brains from BALB/c mice, frozen in liquid nitrogen immediately after surgical extraction, were stored at −80 °C. Sections with a 10 μm thickness were cut using a cryostat microtome CM1850 (Leica Microsystems, Germany) at −20 °C and thaw mounted onto standard microscopic glass slides. A series of sections from the same brain was stained with BB7 or covered with classical matrices by sublimation in a glass sublimation chamber (model GPE-1207–030PS, GPE Scientific, United Kingdom). An amount of 200 mg of DHB or DAN was homogeneously spread in the bottom part of the sublimation chamber. The sublimation parameters were 135 °C, pressure ≤ 100 mTorr, 4 min 20 s for DHB. DAN was sublimated at 130 °C, pressure ≤ 100 mTorr for 2 min 20 s.

### BB7 Staining

For BB7 staining, 100 mg of BB7 (dye content 90%) was dissolved in 10 mL of deionized water. The solution was sonicated for 5 min in an ultrasonic bath and decanted prior to staining to remove residual undissolved dye particles. Subsequently, the glass slide with the tissue section was immersed 3 times for 10 s and 3 times for 30 s, for positive and negative mode measurements, respectively. There were 5 s intervals between each immersion. Finally, the glass with the stained tissue section was washed in a beaker with deionized water and dried under a gentle stream of dry air. The BB7 solution could be used for staining multiple tissues. After processing, the used BB7 solution was collected in a waste container. As BB7 can cause serious eye damage, all manipulation with this compound was done by wearing protective gloves and goggles.

### BB7 Spraying

The BB7 solution was prepared by dissolving 5 mg of the dye powder (dye content 90%) in 1 mL of deionized water. An amount of 300 μL of the solution was pipetted into the airbrush pistol (Grafo T1, 0.15 mm, HARDER and STEENBECK, Germany) reservoir and was manually sprayed onto the sample from a distance of 14 cm. The spraying was realized in cycles: approximately 1 s of spraying was followed by 2 s of drying until the solution was consumed. The solution flow was 150 μL·min^–1^.

### MALDI MSI

MALDI MS and MSI data were acquired using an orbital trap (Q Exactive Plus Orbitrap, Thermo Fisher Scientific, Germany) equipped with a subatmospheric (subAP) MALDI source (SubAP/MALDI(ng), MassTech, Inc., USA). Ions were transferred from the source to the MS by a two-stage ion funnel. Voltages V7–V1 applied across the funnel were 350, 270, 150, 120, 90, 85, 10 V and −250, −150, −120, −100, −90, −85, and −10 V in positive and negative modes, respectively. The radio frequency (RF) amplitude confining the ions passing through the ion funnel was 130 V (peak-to-peak). Pressure in the source was kept at 2 Torr, providing the optimal signal of phospholipids and ceramides at *m*/*z* range of 600–900. The mass resolving power was 140,000 (at *m*/*z* 200) in all experiments. If not stated otherwise, the pixel size was set to 50 μm.

A sample was continuously moved at a speed of 3.445 mm·min^–1^ under the stationary frequency-tripled Nd:YAG laser (355 nm, 1000 Hz) with a nearly circular spot with a diameter of ∼15 μm (based on microscopic inspection of ablated spots in a DHB layer). In the positive mode, the laser energy was set to 0.32 and 0.60 μJ·pulse^–1^ for DHB and BB7, respectively. For DAN and BB7 in negative ion mode, 0.36 μJ·pulse^–1^ and 0.90 μJ·pulse^–1^ were used, respectively. The *m*/*z* calibration in positive and negative modes was done using red phosphorus.^43^

### Lipid Identification

The lipids were identified based on the exact measured mass, which was compared to the Lipid Maps database. The cases with measured *m*/*z* error less than 0.001 were considered positive assignments.

## Results and Discussion

### BB7 as a MALDI Matrix

BB7 is a heterocyclic compound with several nitrogen atoms (Figure 1A) stable under normal temperatures and pressures.^44^ Though the absorption maximum is at 615 nm, it also absorbs 355 nm of light emitted by the used Nd:YAG laser (Figure 1B), which is sufficient for MALDI MS experiments. The BB7 absorption spectrum was measured using an Agilent HP 8453 UV–vis spectrometer in a quartz cuvette at a concentration of 8 mg·L^–1^ in deionized water.

**Figure 2:**
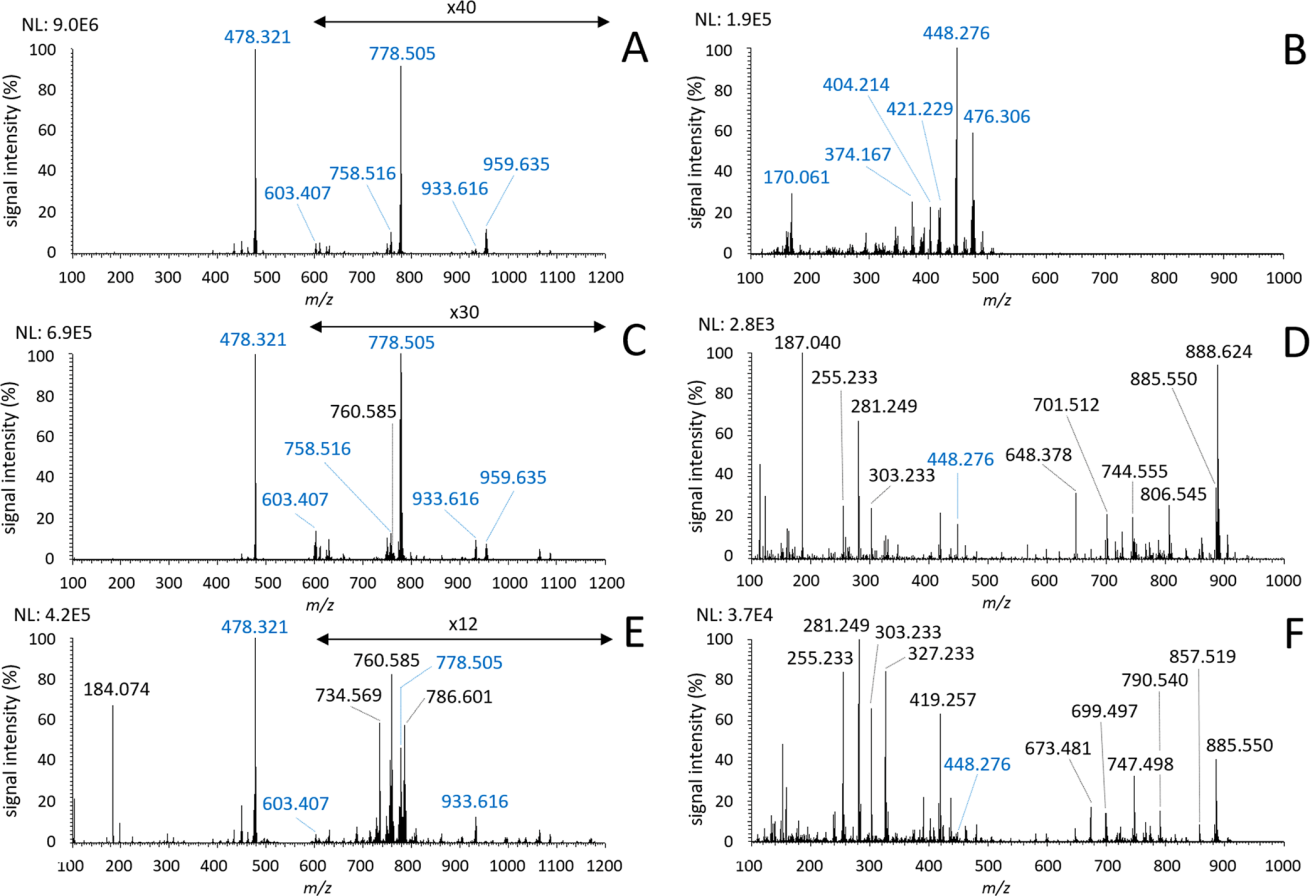
MALDI mass spectra of (A and B): analytical standard BB7; (C and D): dried droplet of brain lipid extract with BB7; and (E and F): mouse brain stained with BB7. Spectra (A, C, and E) were recorded in positive ion mode (signal in the *m*/*z* region 600–1200 was magnified, as indicated) and spectra (B, D, and F) in negative ion mode. Peaks originating from BB7 are marked blue.

The initial experiments were aimed at testing the suitability of BB7 as a MALDI matrix:

For this purpose, spectra were recorded from a solution of BB7 (purity ≥ 95%) with a concentration of 10 mg·mL^–1^ (Figure 2A,B); from a dried droplet of pig brain lipid extract overlaid by a 10 mg·mL^–1^ BB7 solution (Figure 2C,D); and from mouse brain tissue stained by BB7 (Figure 2E,F). In positive ion mode, the dominant signal at *m*/*z* 478.321 can be attributed to the molecular ion of BB7. A few minor signals originating from the LDI of BB7, for example, at *m*/*z* 750.474, 758.516, 776.489, and 778.505, occur at the typical *m*/*z* range of phospholipids and thus could interfere with the lipidomic analysis. For instance, a signal at *m*/*z* 758.516 is close to the signal of the following lipid ions based on a Lipid Maps search: PC 34:2 [M + H]^+^ (*m*/*z* 758.569), PS 34:3 [M + H]^+^ (*m*/*z* 758.497), PS 32:0 [M + Na]^+^ (*m*/*z* 758.494), or PE 34:0 [M + K]^+^ (*m*/*z* 758.510). Similarly, examples of lipid ions with *m*/*z* values close to those of other signals from the BB7 matrix used can also be found. The experimental parameters used allowed baseline separation of peaks with Δ*m*/*z* ≥ 0.02 in this *m*/*z* region. Instruments with lower resolving power (i.e., common TOF MS) would not distinguish these ions at all. In the positive mode spectrum of the dried droplet sample with lipid brain extract, lipids exhibited only minor intensities (Figure 2C); however, in the stained tissue, intense signals of multiple phospholipids, especially PCs, were observed (Figure 2E). In negative mode, the analytical standard of BB7 produced multiple intense signals in the *m*/*z* range 100–500 (Figure 2B). Identical spectra were obtained for BB7 with a dye content of 90% (data not shown). These matrix peaks were strongly suppressed in lipid brain extract droplets overlaid by BB7 and, in the case of the stained brain tissue, most of them disappeared (Figure 2E,F). Thus, very clean spectra of multiple lipid classes with no matrix interference were recorded from the tissue. These results indicated that this compound has great potential as a MALDI MSI matrix, especially in negative ion mode.

The examples of identified lipids belonging to different classes in Tables 1 and 2 are intentionally short, and extended tables with signals assigned to lipids are (Tables S1–S4). As expected, most signals were assigned to phosphatidylcholines (PC) with various degrees of unsaturation in the positive ion mode. The other signals were identified as lysophosphatidylcholines (LPC), diacylglycerols (DG), phosphatidic acids (PAs), phosphatidylethanolamines (PE), and different types of sphingolipids: sphingomyelins (SM), cerebrosides (HexCer) with a single hexose as a headgroup, and gangliosides with multiple sugar residues. Interestingly, the signal ratio of PC [M + H]^+^ to [M + Na]^+^ and [M + K]^+^ ions increased significantly in the BB7-stained tissues compared to BB7-sprayed tissues and tissues with a sublimated layer of DHB. This indicates, that alkali salts are effectively washed out of the tissue during the BB7 staining protocol.

**Table 1:** Examples of Lipids from Different Classes Identified in the Positive Ion Mode from the BB7-Stained Mouse Brain Tissue

Name	Measured Mass	Matched Mass	Delta	Ion
**LPC** 18:1	522.3549	522.3554	0.0005	[M + H]^+^
**DG** 34:1	577.5182	577.5190	0.0008	[M – H_2_O + H]^+^
**PA** 34:1	697.4774	697.4779	0.0005	[M + Na]^+^
**PE** 34:1	718.5391	718.5381	0.0010	[M + H]^+^
**SM (d**36:1)	731.6062	731.6061	–0.0001	[M + H]^+^
**PC** 32:0	734.5693	734.5694	0.0001	[M + H]^+^
**PC** 34:1	760.5851	760.5851	0.0000	[M + H]^+^
**PC** 38:6	806.5688	806.5694	0.0006	[M + H]^+^
**HexCer** 42:2**; O2**	832.6629	832.6637	0.0008	[M + Na]^+^
**NeuACHex2Cer** 38:1**; O2**	1231.7642	1231.7650	0.0008	[M + Na]^+^

**Table 2:** Lipid Classes Identified in the Negative Ion Mode from the BB7-Stained Mouse Brain Tissue

Name	Measured Mass	Matched Mass	Delta	Ion
**FA** 16:0	255.2331	391.2330	–0.0001	[M–H]^−^
**CPA** 16:0	391.2255	391.2255	0.0000	[M–H]^−^
**LPA** 18:1	435.2516	435.2517	0.0001	[M–H]^−^
**LPI** 16:0	571.2884	571.2889	0.0005	[M–H]^−^
**PA** 34:1	673.4813	673.4814	0.0001	[M–H]^−^
**PE** 34:1	716.5237	716.5236	–0.0001	[M–H]^−^
**PC** 34:1	744.5551	744.5549	–0.0002	[M–CH_3_]^−^
**PI** 34:1	835.5344	835.5342	–0.0002	[M–H]^−^
**SHexCer** 42:2**; O2**	888.6237	888.6240	0.0003	[M–H]^−^

Compared to the positive mode, a richer spectrum of lipid classes was observed in the negative ion mode, encompassing fatty acids (FA), cyclic phosphatidic acids (CPA), PAs, PEs, phosphatidylcholines (PC), phosphatidylinositols (PI), and sulfatides (SHexCer). To some extent, cleavage of FAs from the phospholipids, as well as minor fragmentation of phospholipid headgroups leading to the production of PAs during the MALDI process, was observed during the analysis of PC and PE standards (see Figure S1). Most of the ions in negative mode were present according to our expectations as [M – H]^−^ ions. Interestingly, PCs were present in the negative spectra as [M – CH_3_]^−^ ions and also as [M + Cl]^−^ ions, while the peak corresponding to [M + NaCl – H]^−^ was assigned to PEs in the case of the dried droplet analysis of standards. The formation of PC [M – CH_3_]^−^ ions in the stained tissues was also observed using spotting of PC 40:0 standard onto tissue and recording the image of the droplet (see Figure S2).

### MALDI MS Imaging

**Figure 3:**
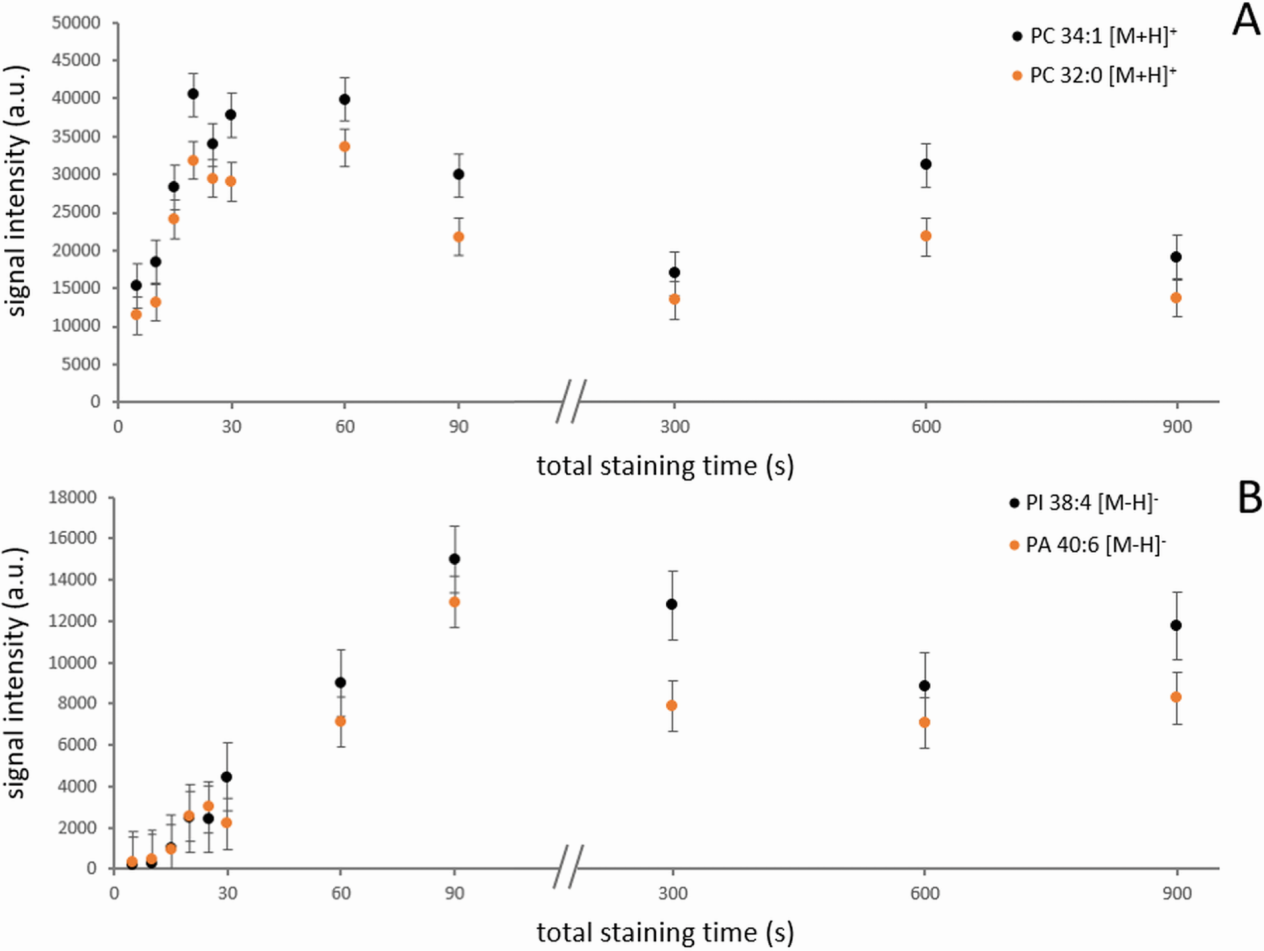
MALDI MS signal intensity of (A) PC 32:0 and PC 34:1 [M + H]^+^ ions in positive ion mode and (B) PA 40:6 and PI 38:4 [M – H]^−^ ions in negative ion mode plotted vs the total BB7 staining time of brain section.

After the initial experiments, we moved on to the MALDI MSI. The mouse brain was selected, as it is the tissue most studied by MSI techniques with a well-known representation and distribution of many lipids. For BB7 staining, different staining times were used in positive and negative modes to obtain the highest intensities of the lipids. The dipping process was optimized by a series of consecutive dips of the single mouse brain tissue into a 10 mg·mL^–1^ BB7 solution. At the beginning, 5-s immersions were carried out. After 30 s of total staining time, the next steps were 60, 90, 300, 600, and 900 s. After each immersion, the edges of the glass were carefully rubbed with a wipe, removing most of the residual dye solution. Then, the tissue itself was dried by a gentle stream of air within 1 min at room temperature. Subsequently, mass spectra were recorded from five lines scanned across the cortex by a laser, each consisting of 10 pixels. Averaged signal intensities of dominant species with homogeneous distribution in the cortex plotted vs the total staining time are shown in Figure 3. Protonated PC 32:0 (*m*/*z* 734.569), PC 34:1 (*m*/*z* 760.585), and deprotonated PA 40:6 (*m*/*z* 747.497), PI 38:4 (*m*/*z* 885.550) were chosen for positive and negative modes, respectively. In positive mode, the plateau with the highest signal was observed for a total staining time of 20–60 s (Figure 3A). In negative mode, the signal gradually increased to 90 s of total staining time (Figure 3B). The use of long staining times >300 s resulted in slightly lower signals. Based on the results, a 30-s staining time was chosen in the case of positive mode: tissue was dipped into BB7 solution three times for 10 s. For negative mode imaging, the tissue was dipped three times for 30 s, giving a total staining time of 90 s. In the final protocol, the tissue was dried only once after the end of the series of immersions. The spraying protocol also required a higher amount of BB7 in the negative mode to obtain the highest signals; however, signal intensities were generally lower in the positive mode and higher in the negative mode, compared to the staining protocol. Optimization of BB7 spraying is described in Figure S3.

**Figure 4:**
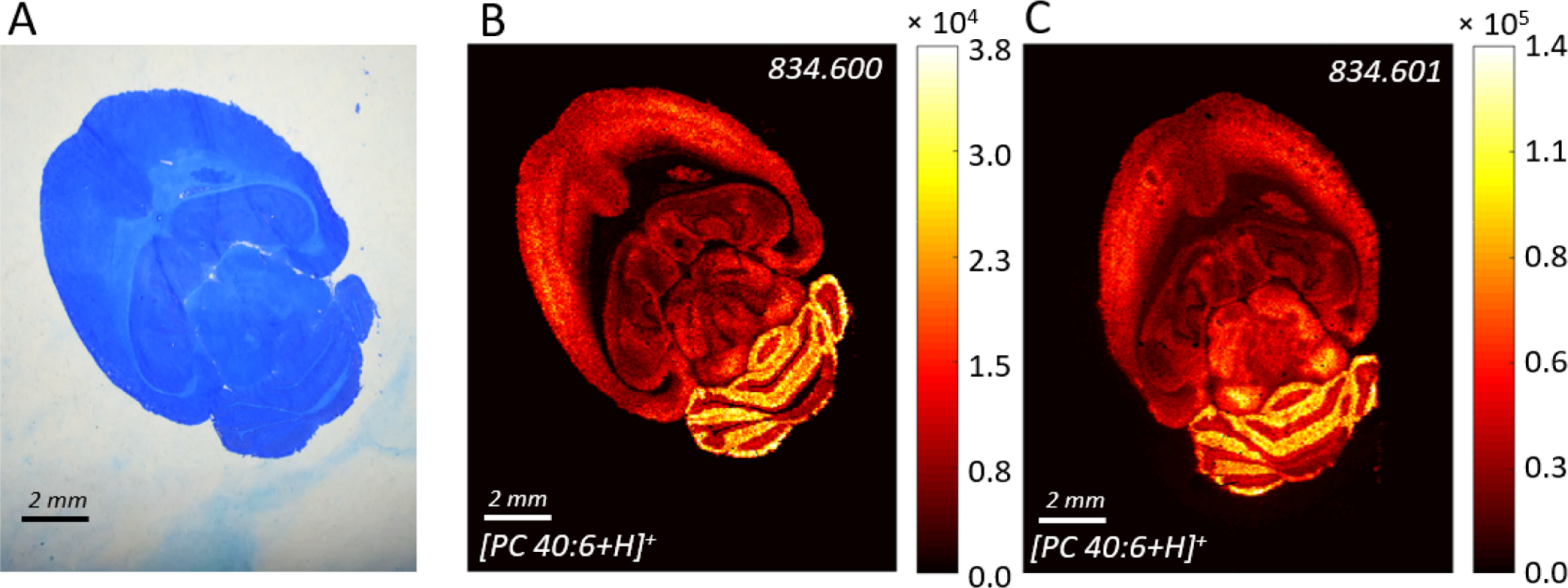
(A) Photo of the BB7-stained mouse brain. MALDI MSI of lipid PC 40:6 recorded from (B) the same BB7-stained section and (C) parallel section with the sublimated DHB layer in positive ion mode.

**Figure 5:**
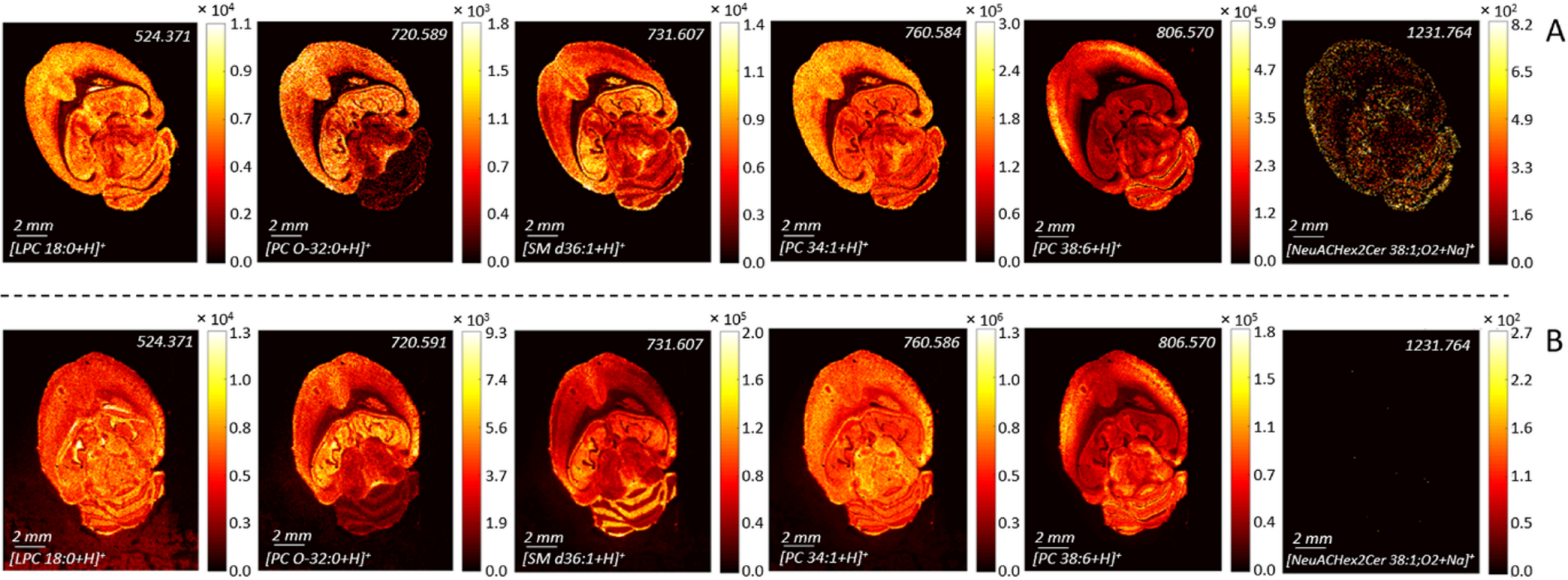
MALDI MSI of the selected lipid (LPC 18:0, PC O-32:0, SM d36:1, PC 34:1, PI O-34:0, and NeuACHex2Cer 38:1;O2) ions in mouse brain tissue recorded by MALDI MSI using (A) BB7 staining and (B) DHB sublimation in positive ion mode.

**Figure 6:**
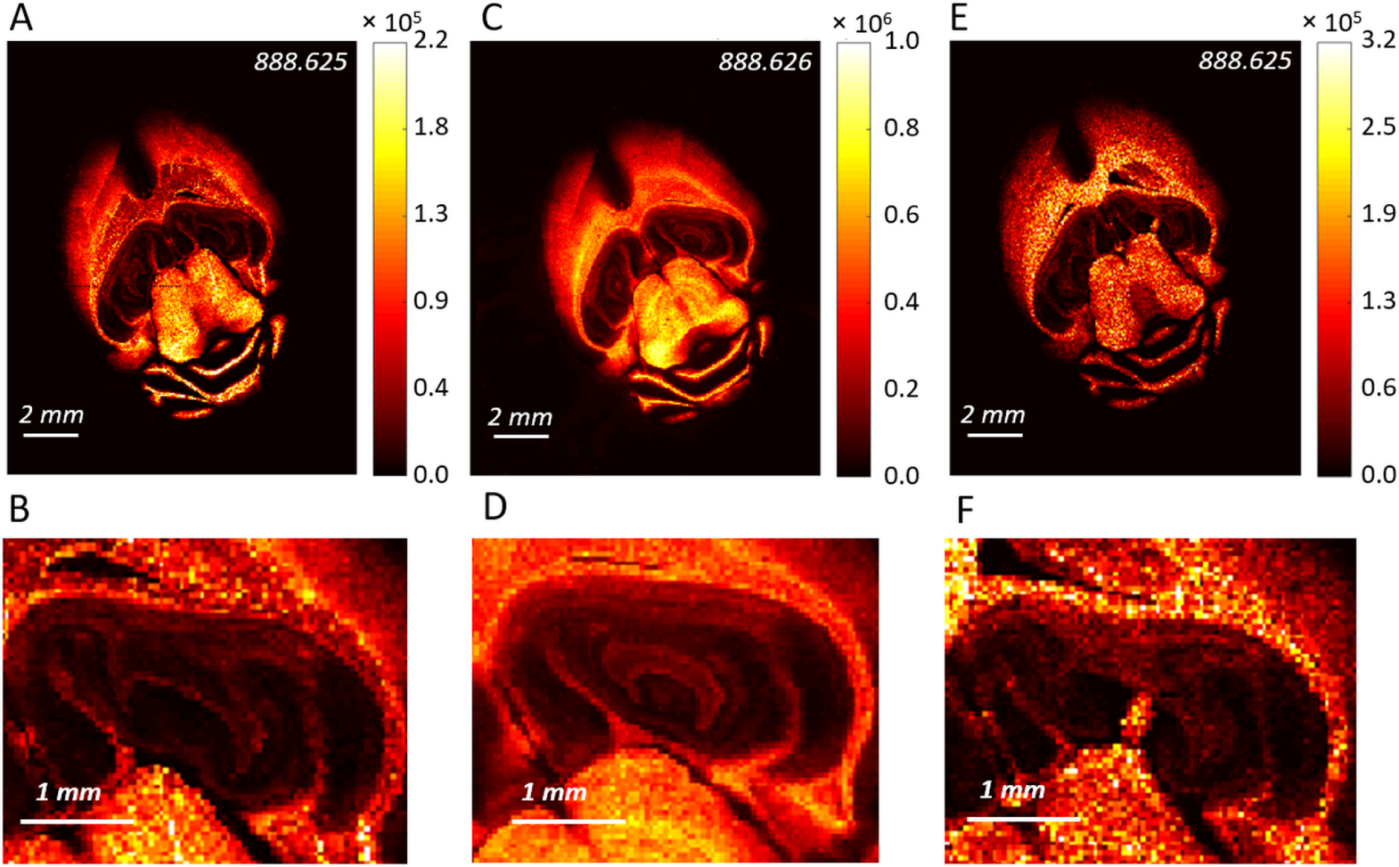
MALDI MSI of SHexCer 42:2;O2 in the mouse brain tissue recorded using (A, B) staining with BB7, (C, D) DAN sublimation, and (E, F) spraying BB7 in the negative mode. (B, D, and F) Show detailed structures in the *hippocampus*, all in the negative mode.

**Figure 7:**
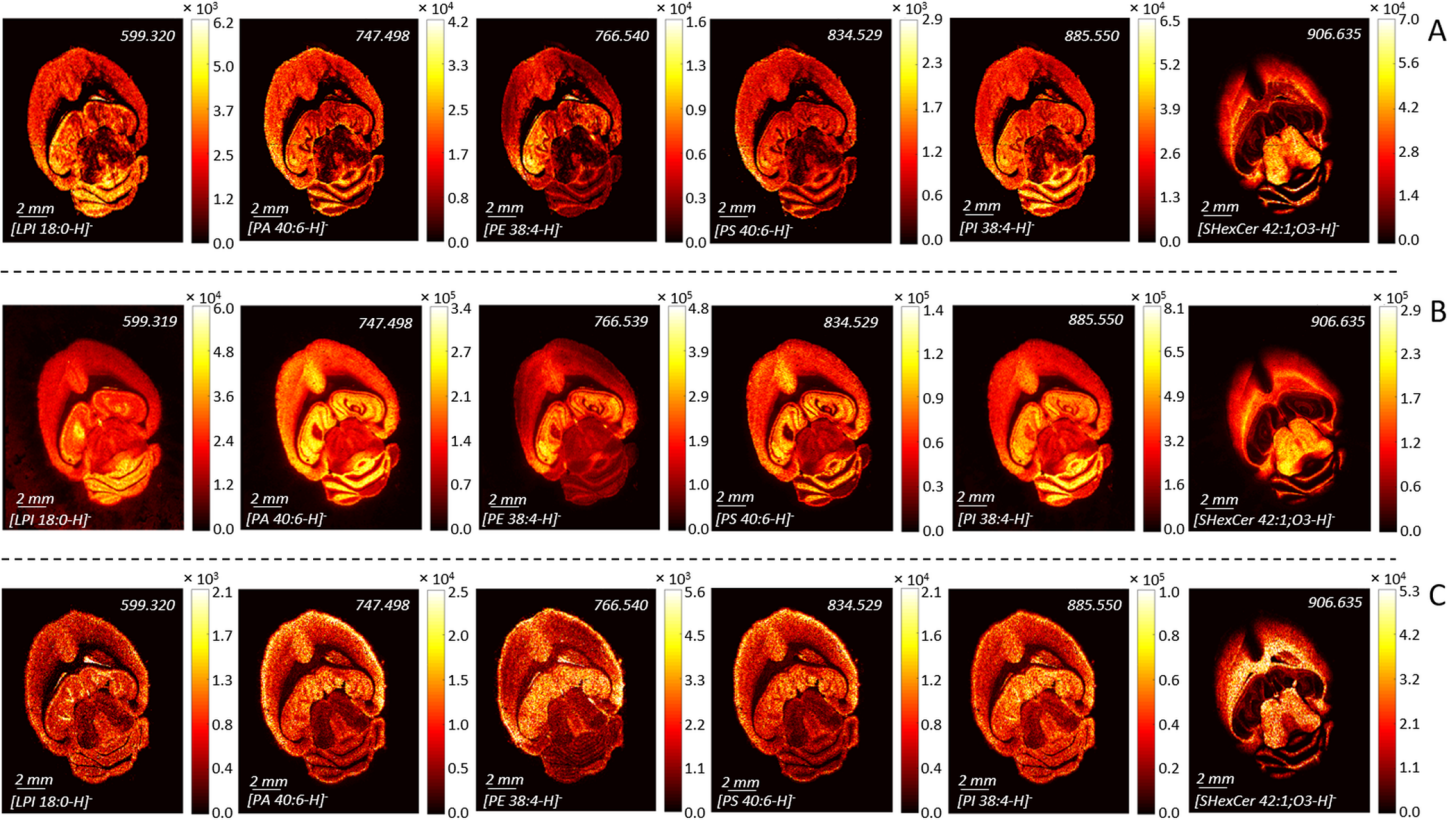
MALDI MSI of selected lipids (LPI 18:0, PA 40:6, PE 38:4, PS 40:6, PI 38:4, and SHexCer 42:1;O3) in the mouse brain tissue recorded by MALDI MSI using (A) BB7 staining, (B) DAN sublimation, and (C) BB7 spraying in negative ion mode.

After optimization of the staining time, MSI experiments were conducted. The parallel mouse brain tissue sections were analyzed using a BB7 matrix (stained as well as sprayed) and sublimated DHB (positive mode) and DAN (negative mode). BB7-stained tissues had several times lower lipid signal intensities compared to tissues coated by DHB and DAN matrices. BB7 staining also provided sharp lipid distribution maps, which were generally comparable to classical matrices (Figures 4–7, S4 and S5); however, certain differences were observed. MS maps obtained for many lipids in positive ion mode (Figures 4, 5), for example, PC O-32:0, or PC 40:6, showed practically the same lipid distribution; however, maps of certain species, such as SM d36:1 or PC 34:1, differed considerably. This can be attributed to several factors. First and most apparent is the uneven distribution of the BB7 matrix observed after visual control of the stained tissues, which is most distinctive in the molecular and granular layer of the *cerebellum* with different densities of neurons (see Figure S6A). BB7 binds to the minor groove of DNA, which makes the neuron nuclei appear as darker blue spots in the stained tissues.^45^ The effect of uneven matrix application can, in theory, be mitigated by image normalization. Though obtained maps of certain lipids (for example, PC 34:1, PC 38:6) after the total ion current (TIC) normalization resembled the corresponding maps with classical matrices more closely, TIC normalization did not have the same positive effect on others (see Figure S7). Other reasons for map differences can be the utilization of close parallel sections and the different magnitudes of suppression levels using two entirely different matrices. The advantage of the BB7 matrix over DHB is the sharpness of the generated maps and the fact that there is no lipid delocalization outside the borders of the tissue, which is seen when DHB is used (Figure 5B). Despite lower signals, BB7 staining was found to be very reproducible and consistently produced sharp MS images without apparent lipid delocalization (see Figure S8). When discussing the image quality, it is important to note the role of decanting the BB7 solution, removing undissolved BB7 particles that cause artifacts in the MS images (see Figure S9). Filtration of the BB7 solution is not advised as a substantial part of the dissolved BB7 was lost on the filter paper during the process.

Comparable results were also obtained in the negative mode (Figures 6 and 7). Sharp distribution maps of many lipid classes (PAs, PEs, PIs, and sulfatides) with a similar level of detail were obtained using BB7 compared to DAN. This is illustrated by the distribution of SHexCer 42:2;O2, revealing fine structures with sharp edges across the BB7-stained tissue and tissue with the sublimated DAN layer (Figure 6B,D). To assess the magnitude of signal variation caused by an uneven distribution of BB7 on the stained tissue, one of the parallel sections was coated with a homogeneous BB7 layer by spraying. Generally, a different contrast of lipids was observed in the cerebellum and white matter of the *corpus callosum*, indicating considerable suppression effects caused by different BB7 application protocols. Staining provided the highest intensities of many lipids, for example, PA 40:6 or PI 38:4, in the molecular layer of the cerebellum (where there was visually less BB7), which closely copies the images recorded with DAN. The same pattern is observed even using BB7 spraying; however, the highest intensities of PA 40:6 or PI 38:4 are in the hippocampal formation. At this moment, it is hard to estimate which approach displays MS maps more accurately, reflecting the exact content of lipids in the different brain regions. At first glance, the spraying protocol should be more accurate as it provides a more homogeneous matrix coverage, but still, the distribution of many lipids in stained tissue, for example, PA 40:6, matches more closely the maps obtained with DAN. Changes in analyte extraction and/or matrix morphology induced by the physical and chemical properties of the underlying tissue type are the main causes for the observed bias in signal intensities. However, this is a general property of current MALDI MS techniques that do not rely on the utilization of external standards on the tissues. Due to these effects, obtained maps can be significantly different compared to actual lipid contents in the tissue determined, for example, by laser capture microdissection followed by extraction and quantitative HPLC MS, and thus, great care always has to be put into data evaluation.^46^

No visible presence of BB7 crystals on stained tissue visualized under the microscope, and the sharpness of the obtained MS maps holds promise for high-spatial-resolution MSI experiments using tissue staining. To show this potential, MS images of mouse cerebellum structures were recorded with a pixel size of 10 μm, at the limits of the used MALDI interface. Detailed structures of SHexCer 40:1 and PI 36:4 were revealed at the borders of molecular and granular layers of the cerebellum (Figure S10).

An added benefit of BB7 staining is the option of direct histological annotation of the BB7-stained tissue, as illustrated in Figure S6. A histopathology specialist was able to directly annotate various regions in the BB7-stained brain, such as the brain cortex, corpus callosum, molecular and granular layers of the cerebellum, and parts of the hippocampus (dentate gyrus or cornu Ammonis; see Figure S6A). For comparison, one of the parallel sections was stained by classical hematoxylin–eosin staining (Figure S6B). For this purpose, it is advised to take a picture of the stained tissue before the MSI experiment, as the employed laser partially ablates BB7 from the tissue.

## Conclusions

We have introduced a new dye-based MALDI MSI matrix, Basic Blue 7, which is soluble in water and thus can easily be used for staining biological tissues, similar to methods used in classical histology. This opens a new and extremely simple method for MALDI MSI sample preparation. The staining technique does not require any special instrumentation and can be easily adapted and automated, which is a crucial factor in clinical practice. The BB7 staining is usable in positive ion mode, where alkali salts are efficiently washed out of the tissue, leading to a dominant presence of [M + H]^+^ ions. The developed protocol excels especially in the generation of negative ions by proton transfer from the analyte to the matrix, thanks to the basic tertiary amide groups in the molecule. Obtained molecular maps of BB7-stained tissues are very sharp, comparable to maps obtained using sublimated DHB and DAN, without any signs of lipid delocalization or spreading out of the tissue borders. The staining protocol has great potential in high-spatial-resolution imaging, as BB7 is adsorbed on the tissue during staining with no visible crystals, the size of which often limits the spatial resolution in MALDI MSI. A certain limitation of the staining protocol is that it cannot be used for the imaging of water-soluble analytes. Compared to DAN or DHB, BB7 is a relatively hot matrix, inducing in-source fragmentation of certain lipids, and it requires higher laser pulse energy for optimal results. In contrast to DAN, which is a potential carcinogen, working with BB7 is generally safe. However, it is necessary to wear protective goggles as it can cause serious eye damage. Also, the extent of ion suppression effects caused by a variation in the amount of BB7 in the different tissue regions remains to be further characterized. On the other hand, due to the intense blue color, the stained tissues can be easily inspected and even used for direct histology annotation, which simplifies the comparison of ion images with the various tissue regions. Last but not least, there is a wide variety of other triarylmethane dyes to which BB7 belongs, and many of these dyes can be expected to perform in MALDI MSI in a similar way or even better. In the future, this approach can present a shared point between classical and modern histology techniques and facilitate the spreading of MALDI MSI into clinical practice.

## Supporting Information Available

Spectra of PC and PE standards, optimization of BB7 spraying, additional MALDI MS images in positive and negative modes, histological annotation of BB7 and hematoxylin–eosin stained tissue, MALDI MS image of mouse brain stained with BB7 without decantation, high-spatial-resolution MS image of part of a mouse cerebellum, and tables with annotated lipids for BB7, DHB, and DAN matrices (PDF)

## References

[ref1] Schulz S.; Becker M.; Groseclose M. R.; Schadt S.; Hopf C. Advanced MALDI mass spectrometry imaging in pharmaceutical research and drug development. Curr. Opin. Biotechnol. 2019, 55, 51–59. 10.1016/j.copbio.2018.08.003. 30153614

[ref2] Zhou Q. Q.; Fülöp A.; Hopf C. Recent developments of novel matrices and on-tissue chemical derivatization reagents for MALDI-MSI. Anal. Bioanal. Chem. 2021, 413 (10), 2599–2617. 10.1007/s00216-020-03023-7. 33215311 PMC8007514

[ref3] Zhou D.; Guo S.; Zhang M.; Liu Y. J.; Chen T. J.; Li Z. L. Mass spectrometry imaging of small molecules in biological tissues using graphene oxide as a matrix. Anal. Chim. Acta 2017, 962, 52–59. 10.1016/j.aca.2017.01.043. 28231880

[ref4] Yang X. H.; Wu T.; Liu B. X.; Du Y. P.; Li H. Y.; Zhao S. L.; Lu Y. X. Matrix selection for polymer guanidine analysis by MALDI-TOF MS. Int. J. Mass Spectrom. 2013, 356, 1–6. 10.1016/j.ijms.2013.09.010.

[ref5] Baluya D. L.; Garrett T. J.; Yost R. A. Automated MALDI matrix deposition method with inkjet printing for imaging mass spectrometry. Anal. Chem. 2007, 79 (17), 6862–6867. 10.1021/ac070958d. 17658766

[ref6] Gustafsson O. J. R.; Eddes J. S.; Meding S.; McColl S. R.; Oehler M. K.; Hoffmann P. Matrix-assisted laser desorption/ionization imaging protocol for in situ characterization of tryptic peptide identity and distribution in formalin-fixed tissue. Rapid Commun. Mass Spectrom. 2013, 27 (6), 655–670. 10.1002/rcm.6488. 23418145

[ref7] Shafer C. C.; Neumann E. K. Optimized combination of MALDI MSI and immunofluorescence for neuroimaging of lipids within cellular microenvironments. Front. Chem. 2024, 12, 1334209 10.3389/fchem.2024.1334209. 38406559 PMC10884125

[ref8] Tuck M.; Grelard F.; Blanc L.; Desbenoit N. MALDI-MSI Towards Multimodal Imaging: Challenges and Perspectives. Front. Chem. 2022, 10, 904688 10.3389/fchem.2022.904688. 35615316 PMC9124797

[ref9] Ryabchykov O.; Popp J.; Bocklitz T. Fusion of MALDI Spectrometric Imaging and Raman Spectroscopic Data for the Analysis of Biological Samples. Front. Chem. 2018, 6, 257 10.3389/fchem.2018.00257. 30062092 PMC6055053

[ref10] Tobias F.; Hummon A. Considerations for MALDI-Based Quantitative Mass Spectrometry Imaging Studies. J. Proteome Res. 2020, 19, 3620–3630. 10.1021/acs.jproteome.0c00443. 32786684 PMC8221076

[ref11] Hansen R. L.; Lee Y. J. High-Spatial Resolution Mass Spectrometry Imaging: Toward Single Cell Metabolomics in Plant Tissues. Chem. Rec. 2018, 18 (1), 65–77. 10.1002/tcr.201700027. 28685965

[ref12] Bao Z. B.; Yu D.; Fu J. X.; Gu J. C.; Xu J.; Qin L.; Hu H.; Yang C. Y.; Liu W. J.; Chen L. L.; Wu R.; Liu H. Q.; Xu H. L.; Guo H.; Wang L.; Zhou Y. J.; Li Q.; Wang X. D. 2-Hydroxy-5-nitro-3-(trifluoromethyl)pyridine as a Novel Matrix for Enhanced MALDI Imaging of Tissue Metabolites. Anal. Chem. 2024, 96 (13), 5160–5169. 10.1021/acs.analchem.3c05235. 38470972

[ref13] Liu Y. Q.; Chen L. L.; Qin L.; Han M. M.; Li J. M.; Luo F. X.; Xue K.; Feng J. C.; Zhou Y. J.; Wang X. D. Enhanced in situ detection and imaging of lipids in biological tissues by using 2,3-dicyanohydroquinone as a novel matrix for positive-ion MALDI-MS imaging. Chem. Commun. 2019, 55 (83), 12559–12562. 10.1039/C9CC06961E. 31577294

[ref14] Huang P. S.; Huang C. Y.; Lin T. C.; Lin L. E.; Yang E. H.; Lee C. P.; Hsu C. C.; Chou P. T. Toward the Rational Design of Universal Dual Polarity Matrix for MALDI Mass Spectrometry. Anal. Chem. 2020, 92 (10), 7139–7145. 10.1021/acs.analchem.0c00570. 32314914

[ref15] Weissflog J.; Svatos A. 1,8-Di(piperidinyl)-naphthalene - rationally designed MAILD/MALDI matrix for metabolomics and imaging mass spectrometry. RSC Adv. 2016, 6 (79), 75073–75081. 10.1039/C6RA17237G.

[ref16] Dutta T.; Stekly T.; Kucera L.; Lemr K. Dual-polarity MALDI mass spectrometry and imaging of oil binders and fatty acids in artworks using cyanographene as a single matrix. Talanta 2022, 242, 123291 10.1016/j.talanta.2022.123291. 35183981

[ref17] Yang J. H.; Norris J. L.; Caprioli R. Novel vacuum stable ketone-based matrices for high spatial resolution MALDI imaging mass spectrometry. J. Mass Spectrom. 2018, 53 (10), 1005–1012. 10.1002/jms.4277. 30073737

[ref18] Li N.; Wang P.; Liu X. L.; Han C.; Ren W.; Li T.; Li X.; Tao F. Y.; Zhao Z. W. Developing IR-780 as a Novel Matrix for Enhanced MALDI MS Imaging of Endogenous High-Molecular-Weight Lipids in Brain Tissues. Anal. Chem. 2019, 91 (24), 15873–15882. 10.1021/acs.analchem.9b04315. 31718156

[ref19] Arafah K.; Longuespée R.; Desmons A.; Kerdraon O.; Fournier I.; Salzet M. Lipidomics for Clinical Diagnosis: Dye-Assisted Laser Desorption/Ionization (DALDI) Method for Lipids Detection in MALDI Mass Spectrometry Imaging. OMICS: J. Integr. Biol. 2014, 18 (8), 487–498. 10.1089/omi.2013.0175. 24905741

[ref20] Harayama T.; Riezman H. Understanding the diversity of membrane lipid composition. Nat. Rev. Mol. Cell Biol. 2018, 19 (5), 281–296. 10.1038/nrm.2017.138. 29410529

[ref21] Murphy R. C.; Hankin J. A.; Barkley R. M. Imaging of lipid species by MALDI mass spectrometry. J. Lipid Res. 2009, 50, S317–S322. 10.1194/jlr.R800051-JLR200. 19050313 PMC2674737

[ref22] Dufresne M.; Thomas A.; Breault-Turcot J.; Masson J.; Chaurand P. Silver-Assisted Laser Desorption Ionization For High Spatial Resolution Imaging Mass Spectrometry of Olefins from Thin Tissue Sections. Anal. Chem. 2013, 85, 3318–3324. 10.1021/ac3037415. 23425078

[ref23] Wang H.-Y. J.; Post S. N. J. J.; Woods A. S. A minimalist approach to MALDI imaging of glycerophospholipids and sphingolipids in rat brain sections. Int. J. Mass Spectrom. 2008, 278 (2–3), 143–149. 10.1016/j.ijms.2008.04.005. 19956342 PMC2614269

[ref24] Mielczarek P.; Slowik T.; Kotlinska J. H.; Suder P.; Bodzon-Kulakowska A. The Study of Derivatization Prior MALDI MSI Analysis-Charge Tagging Based on the Cholesterol and Betaine Aldehyde. Molecules 2021, 26 (9), 2737 10.3390/molecules26092737. 34066579 PMC8124285

[ref25] Angerer T. B.; Bour J.; Biagi J. L.; Moskovets E.; Frache G. Evaluation of 6 MALDI-Matrices for 10 μm Lipid Imaging and On-Tissue MSn with AP-MALDI-Orbitrap. J. Am. Soc. Mass Spectrom. 2022, 33 (5), 760–771. 10.1021/jasms.1c00327. 35358390 PMC9074099

[ref26] Schiller J.; Süss R.; Arnhold J.; Fuchs B.; Lessig J.; Müller M.; Petkovic M.; Spalteholz H.; Zschörnig O.; Arnold K. Matrix-assisted laser desorption and ionization time-of-flight (MALDI-TOF) mass spectrometry in lipid and phospholipid research. Prog. Lipid Res. 2004, 43 (5), 449–488. 10.1016/j.plipres.2004.08.001. 15458815

[ref27] Fuchs B.; Bischoff A.; Süss R.; Teuber K.; Schürenberg M.; Suckau D.; Schiller J. Phosphatidylcholines and -ethanolamines can be easily mistaken in phospholipid mixtures: a negative ion MALDI-TOF MS study with 9-aminoacridine as matrix and egg yolk as selected example. Anal. Bioanal. Chem. 2009, 395 (8), 2479–2487. 10.1007/s00216-009-3032-1. 19690837

[ref28] Lorkiewicz P.; Yappert M. C. 2-(2-Aminoethylamino)-5-nitropyridine as a basic matrix for negative-mode matrix-assisted laser desorption/ionization analysis of phospholipids. J. Mass Spectrom. 2009, 44 (1), 137–143. 10.1002/jms.1483. 19086041

[ref29] Thomas A.; Charbonneau J. L.; Fournaise E.; Chaurand P. Sublimation of New Matrix Candidates for High Spatial Resolution Imaging Mass Spectrometry of Lipids: Enhanced Information in Both Positive and Negative Polarities after 1,5-Diaminonapthalene Deposition. Anal. Chem. 2012, 84 (4), 2048–2054. 10.1021/ac2033547. 22243482

[ref30] Goodwin R. J. A. Sample preparation for mass spectrometry imaging: Small mistakes can lead to big consequences. J. Proteomics 2012, 75 (16), 4893–4911. 10.1016/j.jprot.2012.04.012. 22554910

[ref31] Li S. L.; Zhang Y. Y.; Liu J. A.; Han J. J.; Guan M.; Yang H.; Lin Y.; Xiong S. X.; Zhao Z. W. Electrospray deposition device used to precisely control the matrix crystal to improve the performance of MALDI MSI. Sci. Rep. 2016, 6, 37903 10.1038/srep37903. 27885266 PMC5122855

[ref32] Gessel M. M.; Norris J. L.; Caprioli R. M. MALDI imaging mass spectrometry: Spatial molecular analysis to enable a new age of discovery. J. Proteomics 2014, 107, 71–82. 10.1016/j.jprot.2014.03.021. 24686089 PMC4104210

[ref33] Velickovic D.; Zhang G.; Bezbradica D.; Bhattacharjee A.; Pasa-Tolic L.; Sharma K.; Alexandrov T.; Anderton C. R.; Response Surface Methodology As a New Approach for Finding Optimal MALDI Matrix Spraying Parameters for Mass Spectrometry Imaging. J. Am. Soc. Mass Spectrom. 2020, 31 (3), 508–516. 10.1021/jasms.9b00074. 32126772 PMC7293970

[ref34] Gemperline E.; Rawson S.; Li L. J. Optimization and Comparison of Multiple MALDI Matrix Application Methods for Small Molecule Mass Spectrometric Imaging. Anal. Chem. 2014, 86 (20), 10030–10035. 10.1021/ac5028534. 25331774 PMC4204912

[ref35] Hankin J.; Barkley R.; Murphy R. Sublimation as a method of matrix application for mass spectrometric imaging. J. Am. Soc. Mass Spectrom. 2007, 18, 1646–1652. 10.1016/j.jasms.2007.06.010. 17659880 PMC2042488

[ref36] Morikawa-Ichinose T.; Fujimura Y.; Murayama F.; Yamazaki Y.; Yamamoto T.; Wariishi H.; Miura D. Improvement of Sensitivity and Reproducibility for Imaging of Endogenous Metabolites by Matrix-Assisted Laser Desorption/Ionization-Mass Spectrometry. J. Am. Soc. Mass Spectrom. 2019, 30 (8), 1512–1520. 10.1007/s13361-019-02221-7. 31044355

[ref37] Goodwin R. J.; MacIntyre L.; Watson D. G.; Scullion S. P.; Pitt A. R. A solvent-free matrix application method for matrix-assisted laser desorption/ionization imaging of small molecules. Rapid Commun. Mass Spectrom. 2010, 24 (11), 1682–1686. 10.1002/rcm.4567. 20486266

[ref38] Puolitaival S.; Burnum K.; Cornett D.; Caprioli R. Solvent-free matrix dry-coating for MALDI Imaging of phospholipids. J. Am. Soc. Mass Spectrom. 2008, 19, 882–886. 10.1016/j.jasms.2008.02.013. 18378160 PMC2696184

[ref39] Vergeiner S.; Schafferer L.; Haas H.; Müller T. Improved MALDI-TOF Microbial Mass Spectrometry Imaging by Application of a Dispersed Solid Matrix. J. Am. Soc. Mass Spectrom. 2014, 25 (8), 1498–1501. 10.1007/s13361-014-0923-y. 24894842

[ref40] Lewis L. M.; Indig G. L. Solvent effects on the spectroscopic properties of triarylmethane dyes. Dyes Pigm. 2000, 46 (3), 145–154. 10.1016/S0143-7208(00)00049-8.

[ref41] Hemalatha S.; Rajagobalan B.; Geethakrishnan T. Fabrication, Characterization of Basic Blue 7 Dye-Doped PVA Films and Their Third-Order Nonlinear Optical Properties. J. Fluoresc. 2023, 33, 2295–2304. 10.1007/s10895-023-03228-w. 37036629

[ref42] Koktavá M.; Valášek J.; Bezdeková D.; Prysiazhnyi V.; Adamová B.; Beneš P.; Navrátilová J.; Hendrych M.; Vlček P.; Preisler J.; Bednařík A. Metal Oxide Laser Ionization Mass Spectrometry Imaging of Fatty Acids and Their Double Bond Positional Isomers. Anal. Chem. 2022, 94 (25), 8928–8936. 10.1021/acs.analchem.2c00551. 35713244

[ref43] Sládková K.; Houska J.; Havel J. Laser desorption ionization of red phosphorus clusters and their use for mass calibration in time-of-flight mass spectrometry. Rapid Commun. Mass Spectrom. 2009, 23, 3114–3118. 10.1002/rcm.4230. 19714708

[ref44] Arunsankar N.; Prabakaran A.; Saravanan P.; Vimalan M.; Jeyaram S. Solvent Media on Nonlinear Optical Properties of Triarylmethane Dye via Facile Z-Scan Method. J. Fluoresc. 2023, 1–8. 10.1007/s10895-023-03529-0. 38051401

[ref45] Cong W.; Chen M.; Zhu Z.; Liu Z.; Nan J.; Ye W.; Ni M.; Zhao T.; Jin L. A shortcut organic dye-based staining method for the detection of DNA both in agarose and polyacrylamide gel electrophoresis. Analyst 2013, 138, 1187–1194. 10.1039/c2an36079a. 23296513

[ref46] Eiersbrock F.; Orthen J.; Soltwisch J. Validation of MALDI-MS imaging data of selected membrane lipids in murine brain with and without laser postionization by quantitative nano-HPLC-MS using laser microdissection. Anal. Bioanal. Chem. 2020, 412, 6875–6886. 10.1007/s00216-020-02818-y. 32712813 PMC7496020

