# Supplementary material for: Staining Tissues with Basic Blue 7: A New Dual-Polarity Matrix for MALDI Mass Spectrometry Imaging
Source: Anal Chem. 2025 Jan 30;97(5):2828–36. doi: 10.1021/acs.analchem.4c05244 (PMC11822741; doi:10.1021/acs.analchem.4c05244)
Supplement: Supplementary file 1 — ac4c05244_si_001.pdf [file ac4c05244_si_001.pdf]

Supporting information for

Staining Tissues with Basic Blue 7: A New Dual-Polarity Matrix for MALDI Mass Spectrometry Imaging

Michal Javorek[1], Michal Hendrych[2, 3], Kateřina Ondráková[1], Jan Preisler[1], Antonín Bednařík[1]*

[1] Department of Chemistry, Faculty of Science, Masaryk University, 625 00 Brno, Czech Republic

[2] First Department of Pathology, St. Anne's University Hospital, 602 00 Brno, Czech Republic

[3] First Department of Pathology, Faculty of Medicine, Masaryk University, 625 00 Brno, Czech Republic

Correspondence author:

Antonín Bednařík

Department of Chemistry, Faculty of Science, Masaryk University

Kamenice 5, 625 00 Brno, Czech Republic

email: bednarik@mail.muni.cz

tel.: +420 549 49 6779

Table of contents

Mass spectra of PC and PE standards 3
MALDI MSI of PC 40:0 standard spotted onto the tissue 3
Optimization of BB7 spraying 4
MALDI MSI in positive mode 5
MALDI MSI in negative mode 6
Histological annotation 7
MALDI MS images from BB7 stained tissues with and without TIC normalization 8
Reproducibility of BB7 staining MALDI MSI experiments 9
Mouse brain stained without decantation of BB7 solution 10
Annotation of lipids 12
Positive ion mode 13
Negative ion mode 17

Mass spectra of PC and PE standards

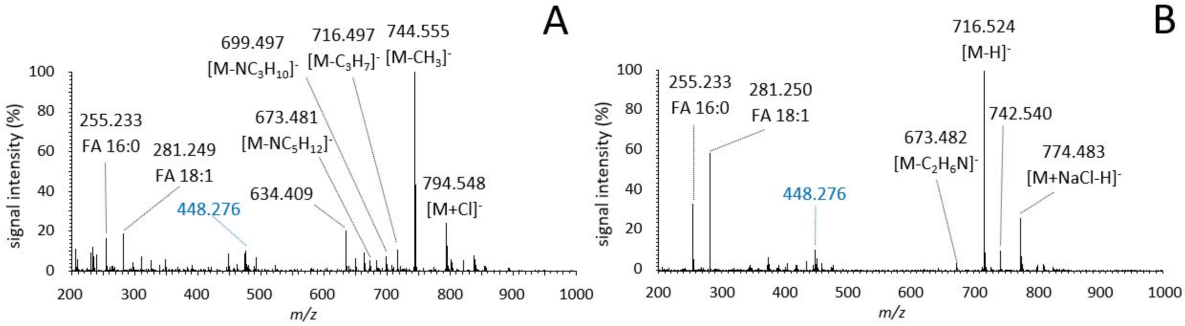

Figure S1: MALDI mass spectra of A) PC 34:1 and B) PE 34:1 standards recorded in negative mode from dried droplets overlaid by BB7 matrix. Peak originating from BB7 is marked blue.

MALDI MSI of PC 40:0 standard spotted onto the tissue

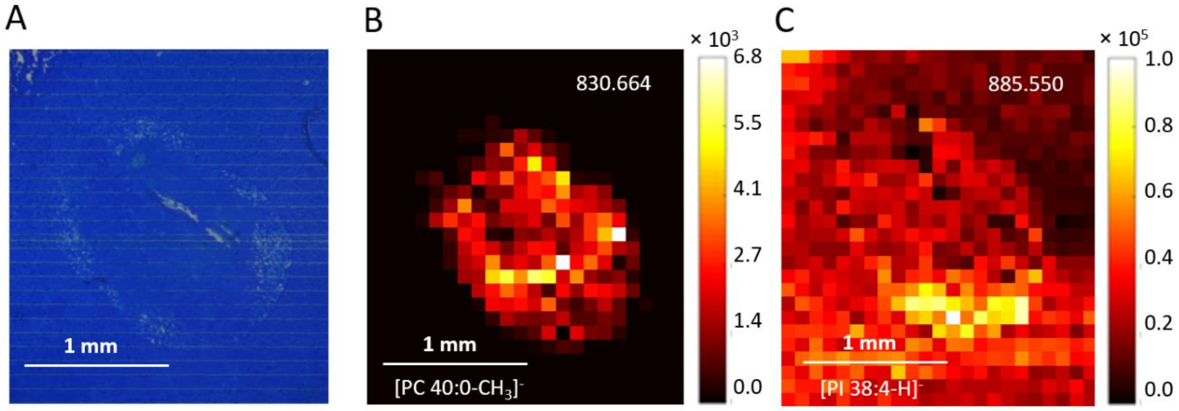

Figure S2 : A) Photo of a dried droplet of PC 40:0 standard (10 mg.mL^{-1} in ethanol) spotted onto the mouse brain tissue after MSI experiment (tissue was stained after the standard spotting) and corresponding MS images of B) PC 40:0 $[M\text{-}CH_3]^-$ and C) PI 38:4 $[M\text{-}H]^-$ ions. Image was recorded with 100 μm pixel size.

Optimization of BB7 spraying

The matrix solution was prepared and applied as described in the chapter "*BB7 spraying"* in the main text. Up to 2 mL of this solution was consecutively applied to the mouse brain tissue. For optimization, the signals of protonated PC 32:0 (*m/z* 734.569), PC 34:1 (*m/z* 760.585) and deprotonated PA 40:6 (*m/z* 747.497), PI 38:4 (*m/z* 885.550) were plotted vs. the total volume of sprayed BB7 solution in the positive and negative modes, respectively (Figure S7). The same data processing was applied as in the case of BB7 staining.

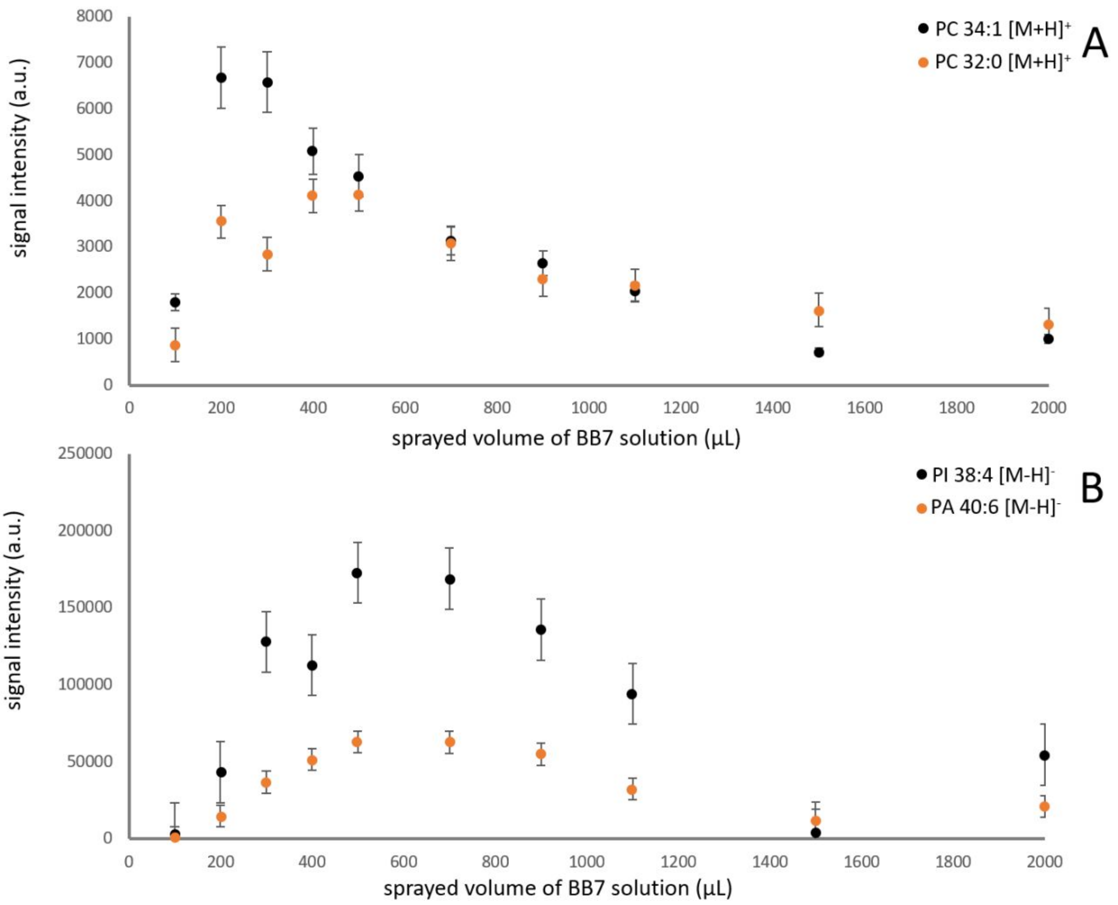

Figure S3: MALDI MS signal intensity of A) PC 32:0 $[M+H]^+$ and PC 34:1 $[M+H]^+$ in positive ion mode and B) PA 40:6 $[M-H]^-$ and PI 38:4 $[M-H]^-$ in negative ion mode plotted vs. total spraying amount of BB7 solution on the brain tissue.

MALDI MSI in positive mode

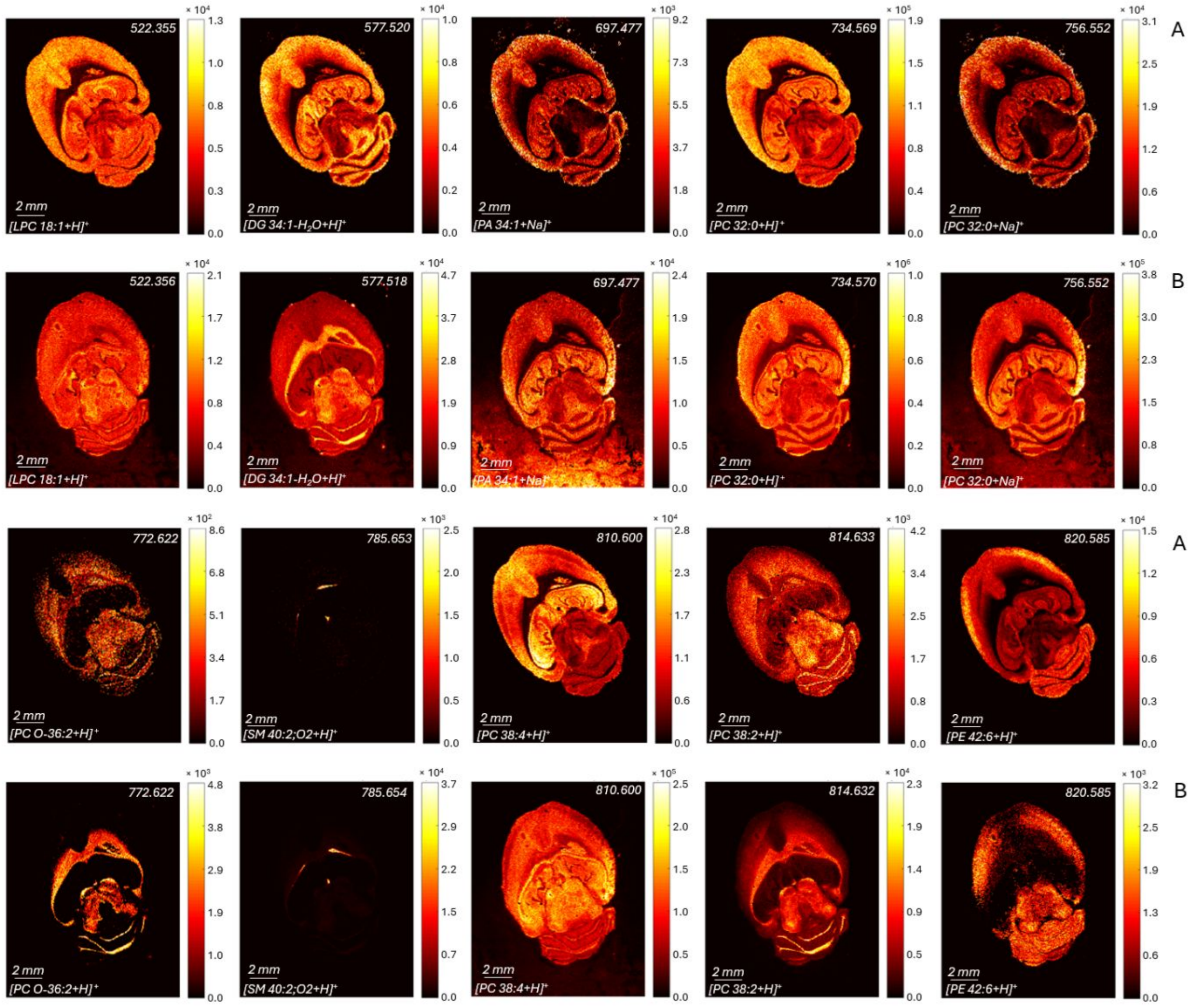

Figure S4: MALDI images of selected lipids recorded in positive ion mode using A) BB7 - staining and B) DHB sublimation (the visualized ions are marked with * in Tables S1 and S2).

MALDI MSI in negative mode

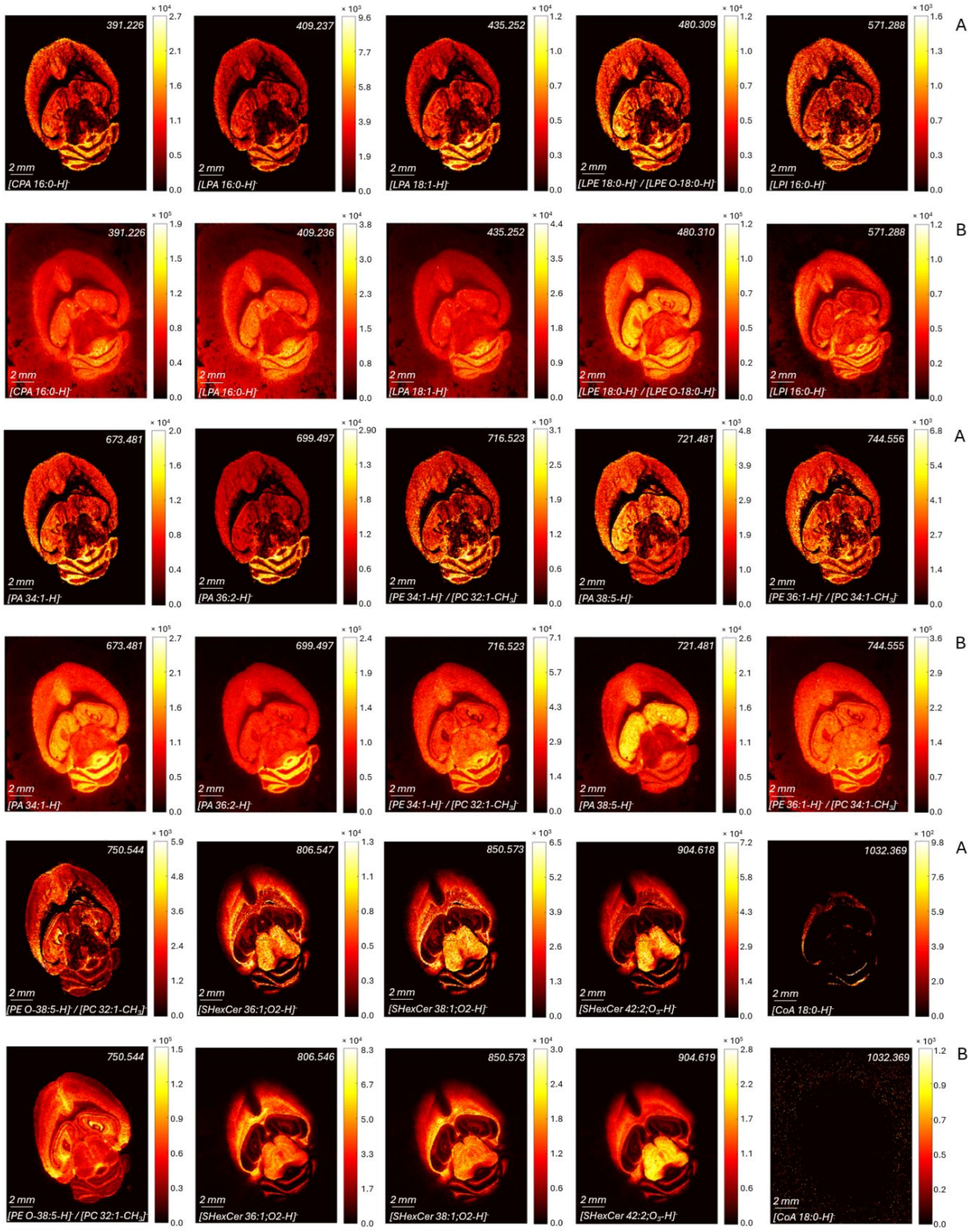

Figure S5: MALDI images of selected lipids in negative ion mode recorded using A) BB7 – staining and B) DAN sublimation (the visualized ions are marked with * in Tables S3 and S4).

Histological annotation

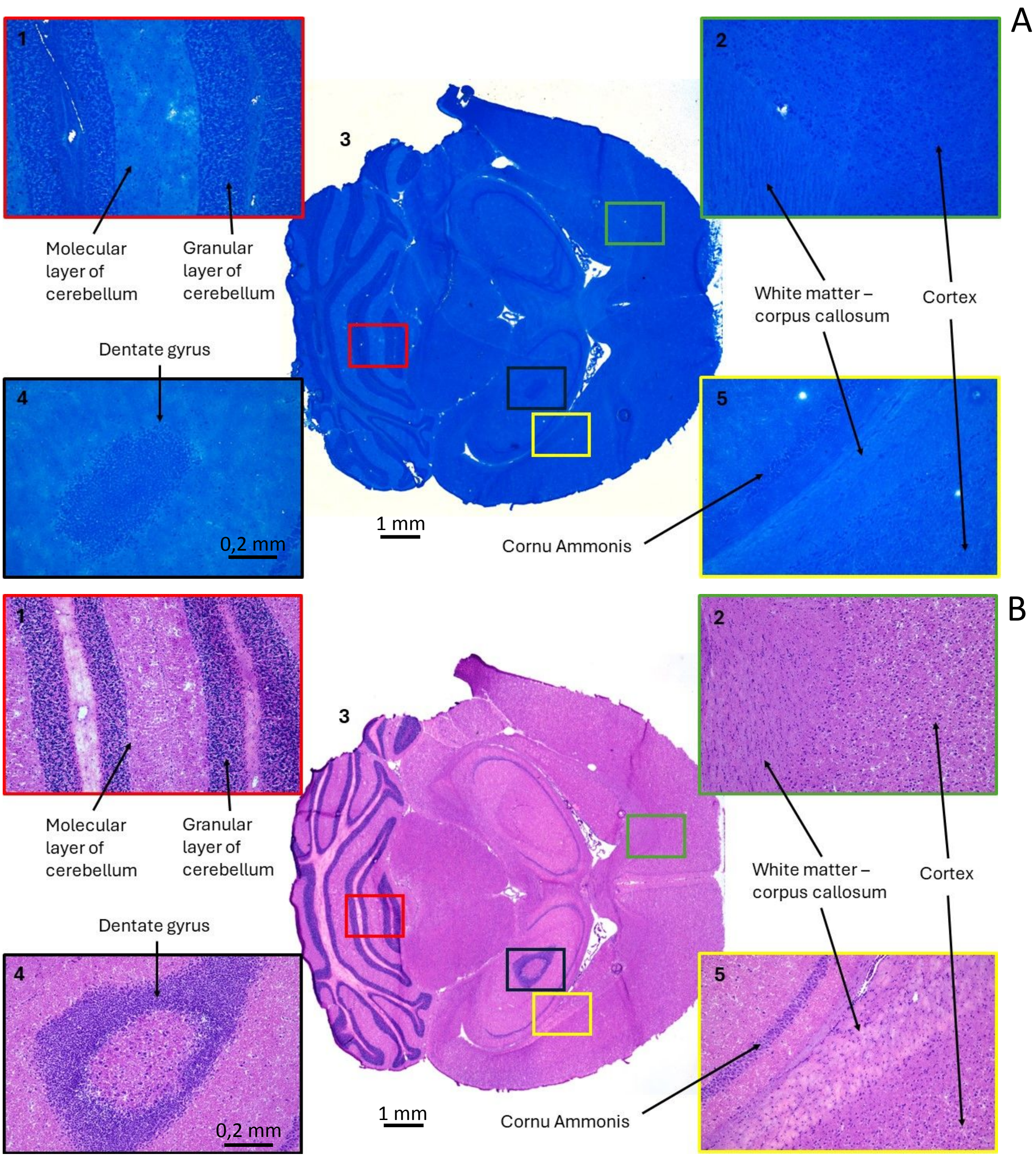

Figure S6: Whole mouse brain section stained by A) BB7 and B) hematoxylin-eosin. 1 - the detailed image of the cerebellum, 2 - the detailed image of the cortex, 3 - the whole mouse brain section, 4 - the detailed image of the hippocampus, 5 - the detailed image of the hippocampus and the cortex.

MALDI MS images from BB7 stained tissues with and without TIC normalization

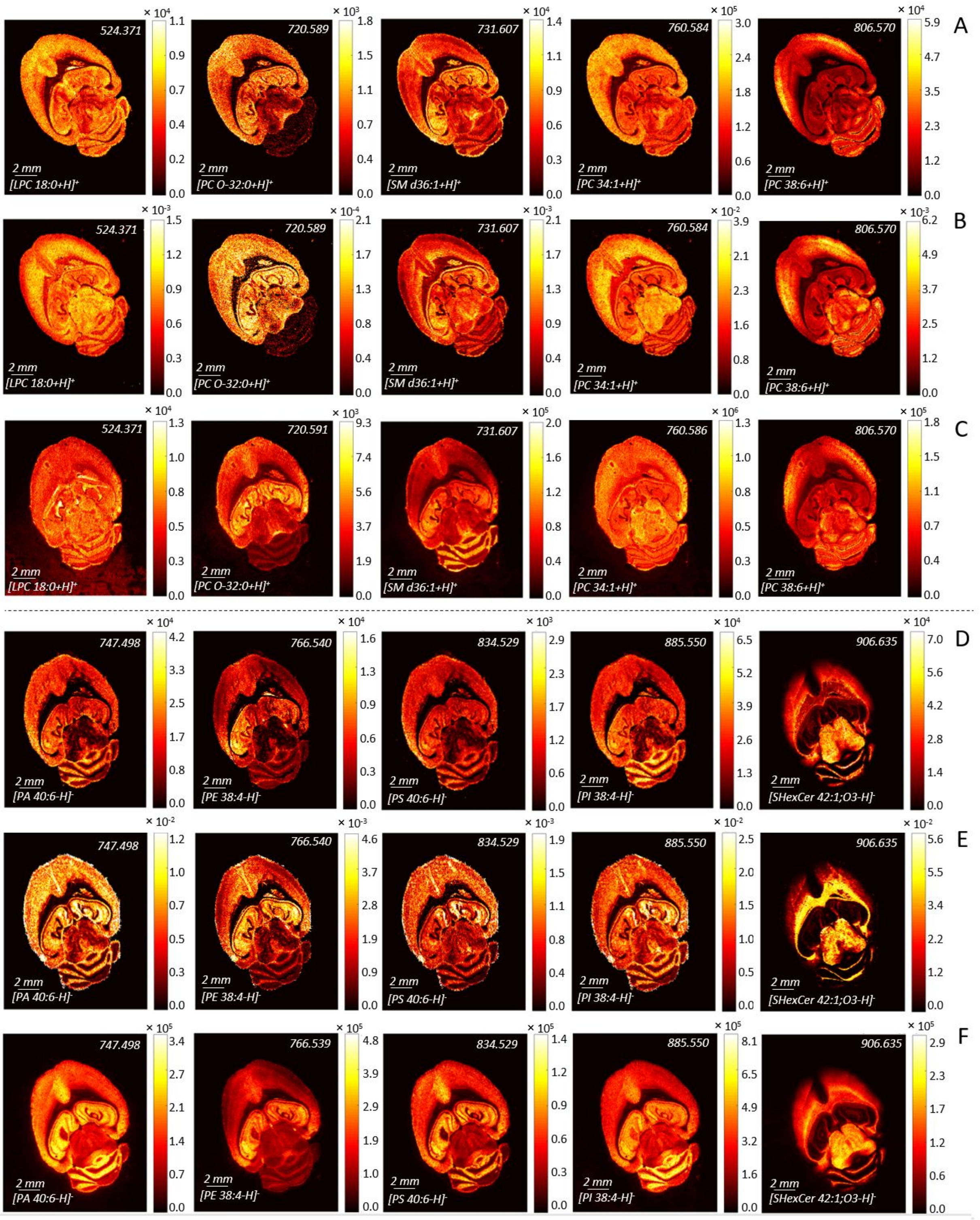

Figure S7: MS images of selected lipids in A-C) positive and D-F) negative modes. BB7 staining A, D) without and B, E) with TIC normalization, C) DHB and F) DAN sublimation without TIC normalization. TIC-generated artefacts in normalized images in negative mode outside the tissue (E) were removed by ROI selection.

Reproducibility of BB7 staining MALDI MSI experiments

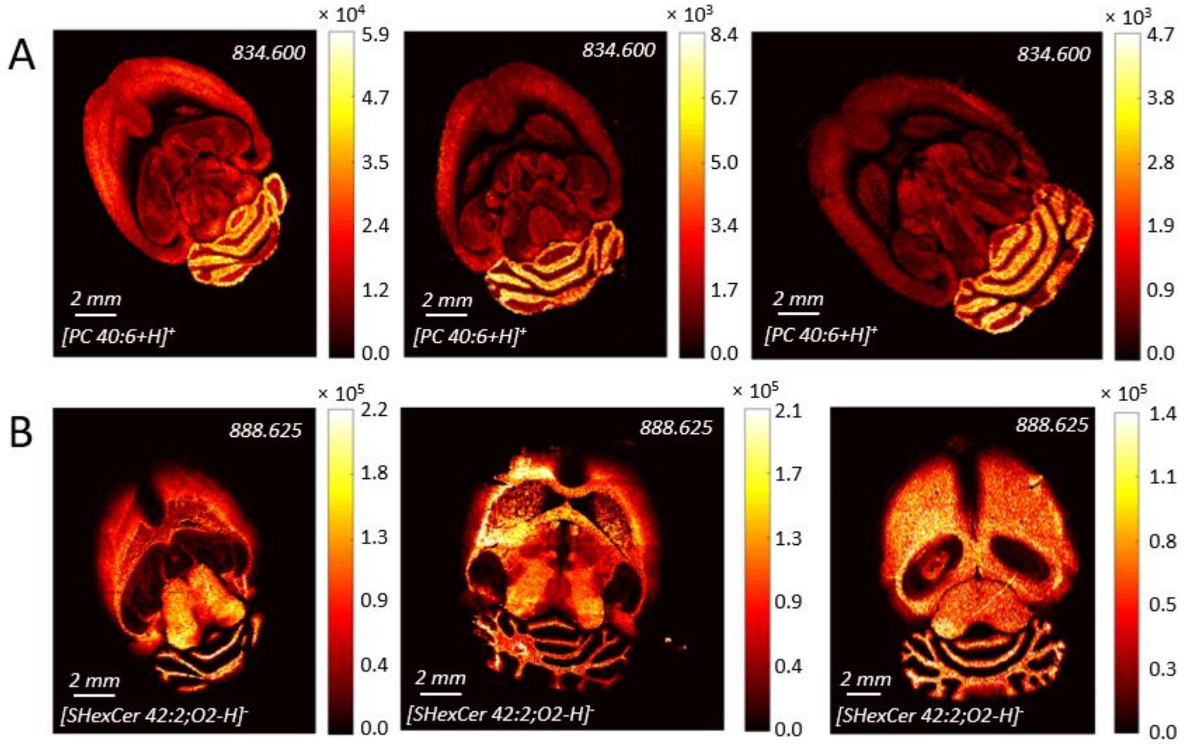

Figure S8: MALDI MS images of A) lipid PC 40:6 $[M+H]^+$ in positive ion mode and B) SHexCer 42:2;O2 $[M-H]^-$ in negative ion mode obtained from 3 different brain tissue sections stained by BB7 matrix.

Mouse brain stained without decantation of BB7 solution

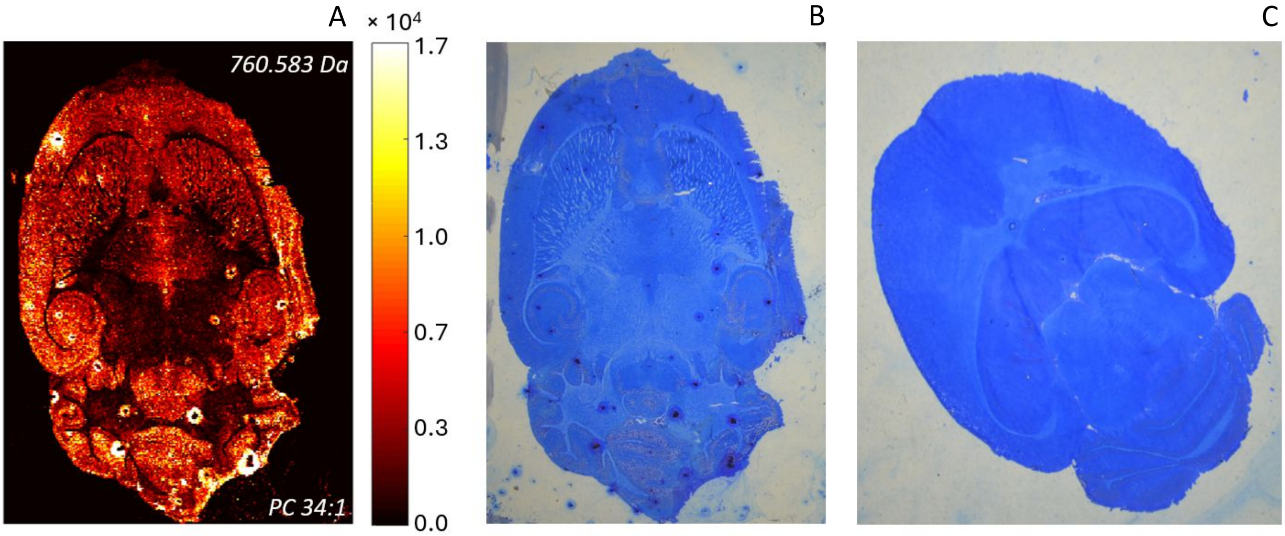

Figure S9: A) MALDI MS image of PC 34:1 and B) a photo of a brain section with artifacts after staining without decantation of BB7 solution. C) A photo of a brain section stained after decantation of BB7 solution.

High-resolution MALDI MS image of the cerebellum

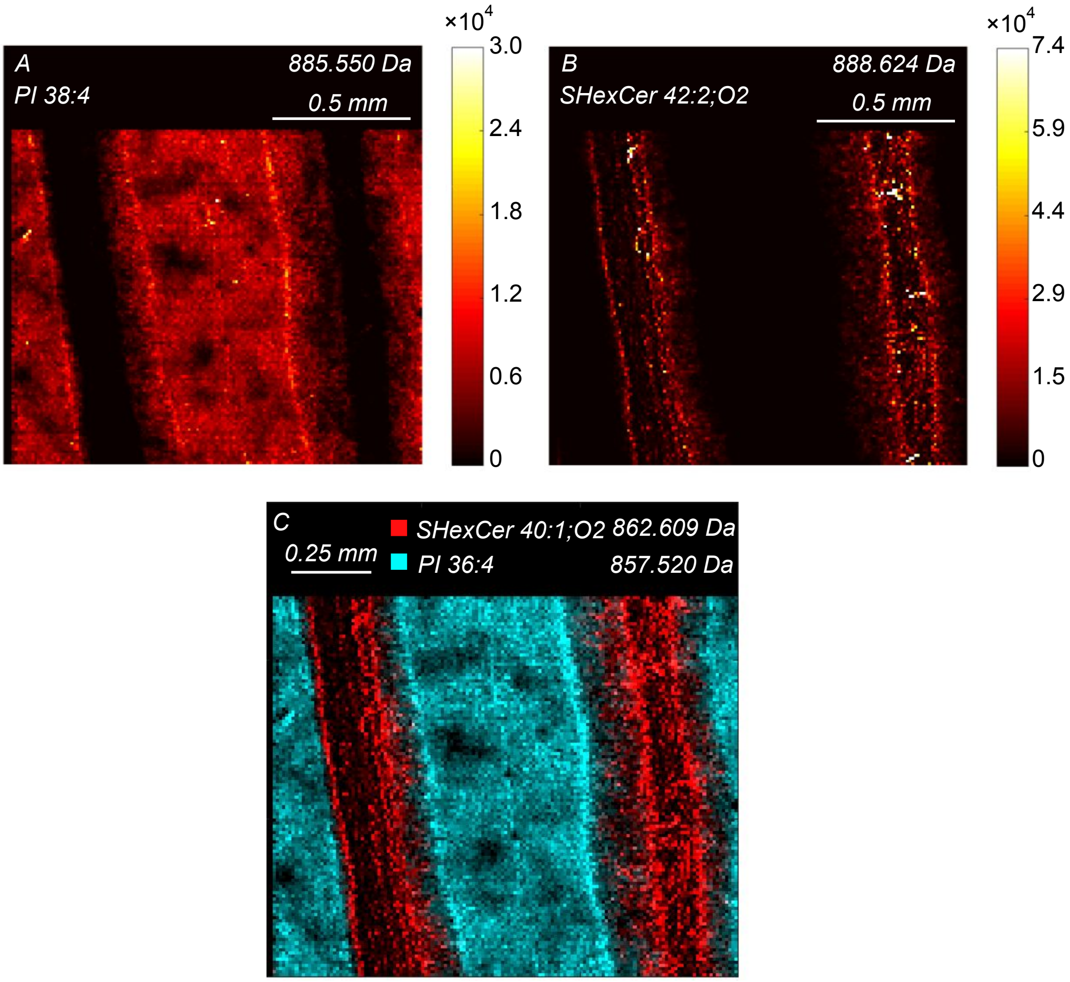

Figure S10: High-resolution MALDI MSI of A) PI 38:4, B) SHexCer 42:2;O2, and C) combined image of SHexCer 40:1;O2 and PI 36:4 in the cerebellum of the mouse brain recorded in negative mode as $[M-H]^-$ ions. The measurement parameters were as follows: speed 1.1482 $mm.s^{-1}$, laser energy 0.90 $\mu J.pulse^{-1}$, pixel size 10 μm.

Annotation of lipids

Lipids were identified from mass spectra obtained from MS and MSI measurements using the Lipids Maps database based on the exact mass in the *m/z* range 500 – 1300. An identification was considered a match of measured and theoretical *m/z* values with an error less than 0.001. Also, we had chosen a signal intensity threshold in the average spectra of 10 a.u. High-intensity signals of well-known lipid ions (PC 34:1 $[M+H]^+$ ion at *m/z* 760.5851 and PI 38:4 $[M-H]^-$ ion at *m/z* 885.5499 in positive and negative modes, respectively) were used for internal *m/z* calibration of the spectra for the best mass accuracy. Lipids with odd number of carbon atoms in the sidechains were ruled out from the comparison, as they are relatively rare in the mammalian tissues. Using this approach on BB7 stained tissues, 322 and 244 lipids were annotated in the positive and negative modes, respectively. For comparison, 990 and 261 lipids were annotated using the same criteria for DHB (positive mode) and DAN (negative mode) matrices, respectively. The higher number of identified signals in positive mode is related to the presence of $[M+Na]^+$ and $[M+K]^+$ ions in addition to $[M+H]^+$, especially in tissue with sublimated DHB layer. In the case of BB7 staining sodium and pottasium ions were washed out effectively. BB7 stained tissue spectra showed the total number of peaks in the *m/z* range 500 – 1300, typically ≥7000 and ≥14000 in positive and negative modes, respectively. After applying the signal intensity threshold, these numbers were cut down to ~2200 in both modes, indicating the probable presence of many so far unannotated, often low-abundance lipid species. Please note that the numbers above are just for a rough comparison of the matrix performance; deep structural characterization of the annotated lipids was not performed.

Positive ion mode

Measured	Matched Mass	Delta	Lipid	Formula	Ion	Intensity
494.3242	494.3241	-0.0001	LPC 16:1	$C_{24}H_{48}NO_7P$	$[M+H]^+$	+++
494.3607	494.3605	-0.0002	LPE O-20:1	$C_{25}H_{52}NO_6P$	$[M+H]^+$	+++
496.3397	496.3398	+0.0001	LPC 16:0	$C_{24}H_{50}NO_7P$	$[M+H]^+$	++++
506.3599	506.3605	+0.0006	LPC O-18:2	$C_{26}H_{52}NO_6P$	$[M+H]^+$	++++
508.3762	508.3761	-0.0001	LPC O-18:1	$C_{26}H_{54}NO_6P$	$[M+H]^+$	+++
510.3551	510.3554	+0.0003	LPE 20:0	$C_{25}H_{52}NO_7P$	$[M+H]^+$	++++
520.3767	520.3761	-0.0006	LPC O-19:2	$C_{27}H_{54}NO_6P$	$[M+H]^+$	+++
522.3554	522.3554	0.0000	LPC 18:1*	$C_{26}H_{52}NO_7P$	$[M+H]^+$	++++
522.3923	522.3918	-0.0005	LPE O-22:0;O	$C_{27}H_{58}NO_7P$	$[M-H_2O+H]^+$	+++
524.3707	524.3711	+0.0004	LPC 18:0	$C_{26}H_{54}NO_7P$	$[M+H]^+$	++++
528.3458	528.3448	-0.0010	LPC 20:3	$C_{28}H_{52}NO_7P$	$[M-H_2O+H]^+$	+++
536.3716	536.3711	-0.0005	LPE 22:1	$C_{27}H_{54}NO_7P$	$[M+H]^+$	+++
538.3859	538.3867	+0.0008	LPE 22:0	$C_{27}H_{56}NO_7P$	$[M+H]^+$	+++
577.5195	577.5190	-0.0005	DG 34:1*	$C_{37}H_{70}O_5$	$[M-H_2O+H]^+$	++++
603.5351	603.5347	-0.0004	DG 36:2	$C_{39}H_{72}O_5$	$[M-H_2O+H]^+$	+++
605.5513	605.5503	-0.0010	DG 36:1	$C_{39}H_{74}O_5$	$[M-H_2O+H]^+$	+++
697.4769	697.4779	+0.0010	PA 34:1*	$C_{37}H_{71}O_8PNa$	$[M+Na]^+$	++++
706.5380	706.5381	+0.0001	PC 30:0	$C_{38}H_{76}NO_8P$	$[M+H]^+$	+++
718.5754	718.5745	-0.0009	PC O-32:1	$C_{40}H_{80}NO_7P$	$[M+H]^+$	+++
720.5894	720.5902	+0.0008	PC O-32:0	$C_{40}H_{82}NO_7P$	$[M+H]^+$	+++
731.6065	731.6061	-0.0004	SM (d36:1)	$C_{41}H_{83}N_2O_6P$	$[M+H]^+$	+++
732.5530	732.5538	+0.0008	PC 32:1	$C_{40}H_{78}NO_8P$	$[M+H]^+$	++++
734.5690	734.5694	+0.0004	PC 32:0*	$C_{40}H_{80}NO_8P$	$[M+H]^+$	+++++
746.6049	746.6058	+0.0009	PC O-34:1	$C_{42}H_{84}NO_7P$	$[M+H]^+$	++++
748.5849	748.5851	+0.0002	PE 36:0	$C_{41}H_{82}NO_8P$	$[M+H]^+$	++++
756.5522	756.5514	-0.0008	PC 32:0*	$C_{40}H_{80}NO_8PNa$	$[M+Na]^+$	++++
758.5693	758.5694	+0.0001	PC 34:2	$C_{42}H_{80}NO_8P$	$[M+H]^+$	++++
760.5843	760.5851	+0.0008	PC 34:1	$C_{42}H_{82}NO_8P$	$[M+H]^+$	+++++
762.6003	762.6007	+0.0004	PC 34:0	$C_{42}H_{84}NO_8P$	$[M+H]^+$	++++
772.6215	772.6215	0.0000	PC O-36:2*	$C_{44}H_{86}NO_7P$	$[M+H]^+$	+++
774.6003	774.6007	+0.0004	PE 38:1	$C_{43}H_{84}NO_8P$	$[M+H]^+$	+++++
776.6160	776.6164	+0.0004	PE 38:0	$C_{43}H_{86}NO_8P$	$[M+H]^+$	++++
781.6187	781.6194	+0,0007	SM 38:1;O2	$C_{43}H_{87}N_2O_6PNa$	$[M+Na]^+$	+++
782.5696	782.5694	-0.0002	PC 36:4	$C_{44}H_{80}NO_8P$	$[M+H]^+$	+++++
784.5856	784.5851	-0.0005	PC 36:3	$C_{44}H_{82}NO_8P$	$[M+H]^+$	++++
785.6533	785.6531	-0.0002	SM 40:2;O2*	$C_{45}H_{89}N_2O_6P$	$[M+H]^+$	+++
786.6002	786.6007	+0.0005	PC 36:2	$C_{44}H_{84}NO_8P$	$[M+H]^+$	++++
788.6162	788.6164	+0.0002	PC 36:1	$C_{44}H_{86}NO_8P$	$[M+H]^+$	+++++
796.5853	796.5851	-0.0002	PE 40:4	$C_{45}H_{82}NO_8P$	$[M+H]^+$	++++
798.5404	798.5410	+0.0006	PC 34:1	$C_{42}H_{82}NO_8PK$	$[M+K]^+$	+++
802.6312	802.6320	+0.0008	PE 40:1	$C_{45}H_{88}NO_8P$	$[M+H]^+$	++++

806.5696	806.5694	-0.0002	PC 38:6	$C_{46}H_{80}NO_8P$	$[M+H]^+$	+++++
808.5852	808.5851	-0.0001	PC 38:5	$C_{46}H_{82}NO_8P$	$[M+H]^+$	++++
810.5999	810.6007	+0.0008	PC 38:4*	$C_{46}H_{84}NO_8P$	$[M+H]^+$	++++
813.6837	813.6844	+0.0007	SM 42:2;O2	$C_{47}H_{93}N_2O_6P$	$[M+H]^+$	+++
814.6326	814.6320	-0.0006	PC 38:2*	$C_{46}H_{88}NO_8P$	$[M+H]^+$	+++
816.6468	816.6477	+0.0009	PC 38:1	$C_{46}H_{90}NO_8P$	$[M+H]^+$	+++
820.5847	820.5851	+0.0004	PE 42:6*	$C_{47}H_{82}NO_8P$	$[M+H]^+$	++++
820.6211	820.6215	+0.0004	PC O-40:6	$C_{48}H_{86}NO_7P$	$[M+H]^+$	+++
824.6155	824.6164	+0.0009	PE 42:4	$C_{47}H_{86}NO_8P$	$[M+H]^+$	++++
832.5857	832.5851	-0.0006	PC 40:7	$C_{48}H_{82}NO_8P$	$[M+H]^+$	++++
832.6629	832.6637	+0.0008	HexCer 42:2;O2	$C_{48}H_{91}NO_8Na$	$[M+Na]^+$	++
834.5999	834.6007	+0.0008	PC 40:6	$C_{48}H_{84}NO_8P$	$[M+H]^+$	++++
848.6160	848.6164	+0.0004	PE 44:6	$C_{49}H_{86}NO_8P$	$[M+H]^+$	++++
908.5655	908.5647	-0.0008	Am-Hex-PE 38:6	$C_{49}H_{84}NO_{13}P$	$[M+H-H_2O]^+$	+++
926.5757	926.5753	-0.0004	Am-Hex-PE 38:6	$C_{49}H_{84}NO_{13}P$	$[M+H]^+$	++
1153.7214	1153.7204	-0.0010	NeuAcHex2Cer 34:1;O2	$C_{57}H_{104}N_2O_{21}$	$[M+H]^+$	+++
1203.7346	1203.7337	-0.0009	NeuAcHex2Cer 36:1;O2	$C_{59}H_{108}N_2O_{21}Na$	$[M+Na]^+$	+++
1231.7648	1231.7650	+0.0002	NeuACHex$_2$Cer 38:1;O2	$C_{61}H_{112}N_2O_{21}Na$	$[M+Na]^+$	+++

Table S1: List of identified lipids with high intensities from the MSI experiment using the BB7 matrix in positive mode. The number of + symbols in the Intensity column indicates the order of magnitude of the signal.

Measured	Matched Mass	Delta	Lipid	Formula	Ion	Intensity
494.3250	494.3241	-0.0009	LPC 16:1	$C_{24}H_{48}NO_7P$	$[M+H]^+$	+++
494.3611	494.3605	-0.0006	LPE O-20:1	$C_{25}H_{52}NO_6P$	$[M+H]^+$	+++
496.3405	496.3398	-0.0007	LPC 16:0	$C_{24}H_{50}NO_7P$	$[M+H]^+$	+++++
506.3606	506.3605	-0.0001	LPC O-18:2	$C_{26}H_{52}NO_6P$	$[M+H]^+$	++++
508.3768	508.3761	-0.0007	LPC O-18:1	$C_{26}H_{54}NO_6P$	$[M+H]^+$	+++
510.3556	510.3554	-0.0002	LPE 20:0	$C_{25}H_{52}NO_7P$	$[M+H]^+$	+++
-	520.3761	-	LPC O-19:2	$C_{27}H_{54}NO_6P$	$[M+H]^+$	
522.3555	522.3554	-0.0001	LPC 18:1*	$C_{26}H_{52}NO_7P$	$[M+H]^+$	++++
-	522.3918	-	LPE O-22:0;O	$C_{27}H_{58}NO_7P$	$[M-H_2O+H)^+$	
524.3711	524.3711	0.0000	LPC 18:0	$C_{26}H_{54}NO_7P$	$[M+H]^+$	++++
528.3445	528.3448	+0.0003	LPC 20:3	$C_{28}H_{52}NO_7P$	$[M-H_2O+H)^+$	+++
536.3706	536.3711	+0.0005	LPE 22:1	$C_{27}H_{54}NO_7P$	$[M+H]^+$	+++
538.3865	538.3867	+0.0002	LPE 22:0	$C_{27}H_{56}NO_7P$	$[M+H]^+$	+++
577.5184	577.5190	+0.0006	DG 34:1*	$C_{37}H_{70}O_5$	$[M-H_2O+H]^+$	++++
603.5355	603.5347	-0.0008	DG 36:2	$C_{39}H_{72}O_5$	$[M-H_2O+H]^+$	++++
605.5503	605.5503	0.0000	DG 36:1	$C_{39}H_{74}O_5$	$[M-H_2O+H]^+$	+++++
697.4769	697.4779	+0.0010	PA 34:1*	$C_{37}H_{71}O_8PNa$	$[M+Na]^+$	++++
706.5382	706.5381	-0.0001	PC 30:0	$C_{38}H_{76}NO_8P$	$[M+H]^+$	++++
718.5742	718.5745	+0.0003	PC O-32:1	$C_{40}H_{80}NO_7P$	$[M+H]^+$	+++
720.5908	720.5902	-0.0006	PC O-32:0	$C_{40}H_{82}NO_7P$	$[M+H]^+$	++++
731.6067	731.6061	-0.0006	SM (d36:1)	$C_{41}H_{83}N_2O_6P$	$[M+H]^+$	+++++
732.5538	732.5538	0.0000	PC 32:1	$C_{40}H_{78}NO_8P$	$[M+H]^+$	+++++
734.5693	734.5694	+0.0001	PC 32:0*	$C_{40}H_{80}NO_8P$	$[M+H]^+$	++++++
746.6057	746.6058	+0.0001	PC O-34:1	$C_{42}H_{84}NO_7P$	$[M+H]^+$	++++
748.5854	748.5851	-0.0003	PE 36:0	$C_{41}H_{82}NO_8P$	$[M+H]^+$	++++
756.5514	756.5514	0.0000	PC 32:0*	$C_{40}H_{80}NO_8PNa$	$[M+Na]^+$	+++++
758.5701	758.5694	-0.0007	PC 34:2	$C_{42}H_{80}NO_8P$	$[M+H]^+$	++++
760.5855	760.5851	-0.0004	PC 34:1	$C_{42}H_{82}NO_8P$	$[M+H]^+$	++++++
762.6011	762.6007	-0.0004	PC 34:0	$C_{42}H_{84}NO_8P$	$[M+H]^+$	+++++
772.6224	772.6215	-0.0009	PC O-36:2*	$C_{44}H_{86}NO_7P$	$[M+H]^+$	+++
774.6012	774.6007	-0.0005	PE 38:1	$C_{43}H_{84}NO_8P$	$[M+H]^+$	++++
776.6163	776.6164	+0.0001	PE 38:0	$C_{43}H_{86}NO_8P$	$[M+H]^+$	+++
781.6187	781.6194	+0.0007	SM 38:1;O2	$C_{43}H_{87}N_2O_6PNa$	$[M+Na]^+$	++++
782.5697	782.5694	-0.0003	PC 36:4	$C_{44}H_{80}NO_8P$	$[M+H]^+$	+++++
784.5851	784.5851	0.0000	PC 36:3	$C_{44}H_{82}NO_8P$	$[M+H]^+$	++++
785.6537	785.6531	-0.0006	SM 40:2;O2*	$C_{45}H_{89}N_2O_6P$	$[M+H]^+$	++++
786.6006	786.6007	+0.0001	PC 36:2	$C_{44}H_{84}NO_8P$	$[M+H]^+$	+++++
788.6169	788.6164	-0.0005	PC 36:1	$C_{44}H_{86}NO_8P$	$[M+H]^+$	+++++
796.5854	796.5851	-0.0003	PE 40:4	$C_{45}H_{82}NO_8P$	$[M+H]^+$	++++
798.5403	798.5410	+0.0007	PC 34:1	$C_{42}H_{82}NO_8PK$	$[M+K]^+$	++++++
802.6312	802.6320	+0.0008	PE 40:1	$C_{45}H_{88}NO_8P$	$[M+H]^+$	++++
806.5698	806.5694	-0.0004	PC 38:6	$C_{46}H_{80}NO_8P$	$[M+H]^+$	++++

808.5854	808.5851	-0.0003	PC 38:5	$C_{46}H_{82}NO_8P$	$[M+H]^+$	++++
810.6004	810.6007	+0.0003	PC 38:4*	$C_{46}H_{84}NO_8P$	$[M+H]^+$	+++++
813.6837	813.6844	+0.0007	SM 42:2;O2	$C_{47}H_{93}N_2O_6P$	$[M+H]^+$	++++
814.6317	814.6320	+0.0003	PC 38:2*	$C_{46}H_{88}NO_8P$	$[M+H]^+$	++++
816.6484	816.6477	-0.0007	PC 38:1	$C_{46}H_{90}NO_8P$	$[M+H]^+$	++++
820.5847	820.5851	+0.0004	PE 42:6*	$C_{47}H_{82}NO_8P$	$[M+H]^+$	+++
-	820.6215	-	PC O-40:6	$C_{48}H_{86}NO_7P$	$[M+H]^+$	
824.6164	824.6164	0.0000	PE 42:4	$C_{47}H_{86}NO_8P$	$[M+H]^+$	++++
-	832.5851	-	PC 40:7	$C_{48}H_{82}NO_8P$	$[M+H]^+$	
832.6629	832.6637	+0.0008	HexCer 42:2;O2	$C_{48}H_{91}NO_8Na$	$[M+Na]^+$	++++
834.6008	834.6007	-0.0001	PC 40:6	$C_{48}H_{84}NO_8P$	$[M+H]^+$	+++++
848.6163	848.6164	+0.0001	PE 44:6	$C_{49}H_{86}NO_8P$	$[M+H]^+$	+++
908.5655	908.5647	-0.0008	Am-Hex-PE 38:6	$C_{49}H_{84}NO_{13}P$	$[M-H_2O+H]^+$	+++
926.5757	926.5753	-0.0004	Am-Hex-PE 38:6	$C_{49}H_{84}NO_{13}P$	$[M+H]^+$	+++
-	1153.7204	-	NeuAcHex2Cer 34:1;O2	$C_{57}H_{104}N_2O_{21}$	$[M+H]^+$	
-	1203:7337	-	NeuAcHex2Cer 36:1;O2	$C_{59}H_{108}N_2O_{21}Na$	$[M+Na]^+$	
-	1231.7650	-	NeuACHex2Cer 38:1;O2	$C_{61}H_{112}N_2O_{21}Na$	$[M+Na]^+$	

Table S2: List of identified lipids with high-intensity signals from the MSI experiment using the DHB matrix in positive mode. The number of + symbols in the Intensity column indicates the order of magnitude of the signal.

Negative ion mode

Measured	Matched Mass	Delta	Lipid	Formula	Ion	Isobaric lipid	Ion	Intensity
301.2176	301.2173	-0.0003	**FA 20:5**	$C_{20}H_{30}O_2$	$[M-H]^-$	*ST 20:2;O2*	$[M-H]^-$	++++
303.2334	303.2330	-0.0004	**FA 20:4**	$C_{20}H_{32}O_2$	$[M-H]^-$	*ST 20:1;O2*	$[M-H]^-$	+++++
327.2334	327.2330	-0.0004	**FA 22:6**	$C_{22}H_{32}O_2$	$[M-H]^-$			+++++
329.2490	329.2486	-0.0004	**FA 22:5**	$C_{22}H_{34}O_2$	$[M-H]^-$	*SFE 22:5*	$[M-H]^-$	+++++
331.2648	331.2643	-0.0005	**FA 22:4**	$C_{22}H_{36}O_2$	$[M-H]^-$	*SFE 22:4*	$[M-H]^-$	+++++
333.2801	333.2799	-0.0002	**FA 22:3**	$C_{22}H_{38}O_2$	$[M-H]^-$	*SFE 22:3, WE 22:3*	$[M-H]^-$	++++
335.2958	335.2956	-0.0002	**FA 22:2**	$C_{22}H_{40}O_2$	$[M-H]^-$	*SFE 22:2, WE 22:2*	$[M-H]^-$	+++
337.3122	337.3112	-0.0010	**FA 22:1**	$C_{22}H_{42}O_2$	$[M-H]^-$	*SFE 22:1, WE 22:1*	$[M-H]^-$	+++
339.3274	339.3269	-0.0005	**FA 22:0**	$C_{22}H_{44}O_2$	$[M-H]^-$	*SFE 22:0, WE 22:0*	$[M-H]^-$	+++
385.2374	385.2384	+0.0010	**ST 24:4;O4**	$C_{24}H_{34}O_4$	$[M-H]^-$			+++
391.2255	391.2255	0.0000	**CPA 16:0***	$C_{19}H_{37}O_6P$	$[M-H]^-$			+++++
393.2410	393.2412	+0.0002	**LPA O-16:1**	$C_{19}H_{39}O_6P$	$[M-H]^-$			+++
409.2368	409.2361	-0.0007	**LPA 16:0***	$C_{19}H_{39}O_7P$	$[M-H]^-$			++++
417.2412	417.2412	0.0000	**CPA 18:1**	$C_{21}H_{39}O_6P$	$[M-H]^-$			+++++
419.2569	419.2568	-0.0001	**CPA 18:0**	$C_{21}H_{41}O_6P$	$[M-H]^-$			+++++
435.2516	435.2517	+0.0001	**LPA 18:1***	$C_{21}H_{41}O_7P$	$[M-H]^-$			++++
437.2673	437.2674	+0.0001	**LPA 18:0**	$C_{21}H_{43}O_7P$	$[M-H]^-$			++++
463.2828	463.2830	+0.0002	**LPA 20:1**	$C_{23}H_{44}O_7P$	$[M-H]^-$			+++
480.3094	480.3096	+0.0002	**LPE 18:0***	$C_{23}H_{48}NO_7P$	$[M-H]^-$	*LPE O-18:1;O LPC O-16:0*	$[M-H]^-$ $[M-CH_3]^-$	++++
571.2884	571.2889	+0.0005	**LPI 16:0***	$C_{25}H_{49}O_{12}P$	$[M-H]^-$			+++
599.3197	599.3202	+0.0005	**LPI 18:0**	$C_{27}H_{53}O_{12}P$	$[M-H]^-$			++++
673.4813	673.4814	+0.0001	**PA 34:1***	$C_{37}H_{70}O_8P$	$[M-H]^-$			++++
699.4969	699.4970	+0.0001	**PA 36:2***	$C_{39}H_{73}O_8P$	$[M-H]^-$			++++
701.5124	701.5127	+0.0003	**PA 36:1**	$C_{39}H_{75}O_8P$	$[M-H]^-$			++++
716.5228	716.5236	+0.0008	**PE 34:1***	$C_{39}H_{76}NO_8P$	$[M-H]^-$	*PC 32:1*	$[M-CH_3]^-$	+++
718.5399	718.5392	-0.0007	**PE 34:0**	$C_{39}H_{78}NO_8P$	$[M-H]^-$	*PC 32:0*	$[M-CH_3]^-$	+++
721.4808	721.4814	+0.0006	**PA 38:5***	$C_{41}H_{71}O_8P$	$[M-H]^-$			+++
723.4973	723.4970	-0.0003	**PA 38:4**	$C_{41}H_{73}O_8P$	$[M-H]^-$			++++
726.5446	726.5443	-0.0003	**PE O-36:3**	$C_{41}H_{78}NO_7P$	$[M-H]^-$	*PC O-34:3*	$[M-CH_3]^-$	+++
728.5598	728.5600	+0.0002	**PE O-36:2**	$C_{41}H_{80}NO_7P$	$[M-H]^-$	*PC O-34:2*	$[M-CH_3]^-$	+++
731.5018	731.5021	+0.0003	**PA O-40:7**	$C_{43}H_{73}O_7P$	$[M-H]^-$			+++
744.5555	744.5549	-0.0006	**PE 36:1***	$C_{41}H_{80}O_8P$	$[M-H]^-$	*PC 34:1*	$[M-CH_3]^-$	+++
747.4970	747.4970	0.0000	**PA 40:6**	$C_{43}H_{73}O_8P$	$[M-H]^-$			++++
750.5441	750.5443	+0.0002	**PE O-38:5***	$C_{43}H_{78}O_7P$	$[M-H]^-$	*PC O-36:5*	$[M-CH_3]^-$	++++
762.5078	762.5079	+0.0001	**PE 38:6**	$C_{43}H_{74}O_8P$	$[M-H]^-$	*PC 36:6*	$[M-CH_3]^-$	++++

766.5397	766.5392	-0.0005	**PE 38:4**	$C_{43}H_{78}NO_8P$	$[M-H]^-$			++++
772.5288	772.5287	-0.0001	**PE O-40:8**	$C_{45}H_{76}NO_7P$	$[M-H]^-$			+++
774.5453	774.5443	-0.0010	**PE P-40:6**	$C_{45}H_{78}NO_7P$	$[M-H]^-$			++++
778.5753	778.5756	+0.0003	**PE O-40:5**	$C_{45}H_{82}NO_7P$	$[M-H]^-$	*PC O-38:5*	$[M-CH_3]^-$	+++
788.5234	788.5236	+0.0002	**PE 40:7**	$C_{45}H_{76}NO_8P$	$[M-H]^-$	*PC 38:7*	$[M-CH_3]^-$	+++
790.5400	790.5392	-0.0008	**PE 40:6**	$C_{45}H_{78}NO_8P$	$[M-H]^-$	*PE O-40:7;O*	$[M-H]^-$	++++
794.5701	794.5705	+0.0004	**PE 40:4**	$C_{45}H_{82}NO_8P$	$[M-H]^-$	PC 38:4	$[M-CH_3]^-$	+++
806.5467	806.5458	-0.0009	**SHexCer 36:1;O2***	$C_{42}H_{81}NO_{11}S$	$[M-H]^-$			++++
816.5540	816.5549	+0.0009	**PE 42:7**	$C_{47}H_{80}NO_8S$	$[M-H]^-$	*PC 40:7*	$[M-CH_3]^-$	+++
822.5416	822.5407	-0.0009	**SHexCer 36:1;O3**	$C_{42}H_{81}NO_{12}S$	$[M-H]^-$			++++
824.5658	824.5658	0.0000	**IPC 36:0;O3**	$C_{42}H_{84}NO_{12}P$	$[M-H]^-$			+++
834.5292	834.5291	-0.0001	**PS 40:6**	$C_{46}H_{78}NO_{10}P$	$[M-H]^-$			++++
834.5778	834.5771	-0.0007	**SHexCer 38:1;O2**	$C_{44}H_{85}NO_{11}S$	$[M-H]^-$			+++
838.5613	838.5604	-0.0009	**PS 40:4**	$C_{46}H_{82}NO_{10}P$	$[M-H]^-$			+++
850.5726	850.5720	-0.0006	**SHexCer 38:1;O3***	$C_{44}H_{85}NO_{12}S$	$[M-H]^-$			+++
857.5195	857.5186	-0.0009	**PI 36:4**	$C_{45}H_{79}O_{13}P$	$[M-H]^-$			++++
862.6094	862.6084	-0.0010	**SHexCer 40:1;O2**	$C_{46}H_{89}NO_{11}S$	$[M-H]^-$			++++
878.6042	878.6033	-0.0009	**SHexCer 40:1;O3**	$C_{46}H_{89}NO_{12}S$	$[M-H]^-$			++++
883.5340	883.5342	+0.0002	**PI 38:5**	$C_{47}H_{81}O_{13}P$	$[M-H]^-$			++++
885.5505	885.5499	-0.0006	**PI 38:4**	$C_{47}H_{83}O_{13}P$	$[M-H]^-$			+++++
888.6245	888.6240	-0.0005	**SHexCer 42:2;O2**	$C_{48}H_{91}NO_{11}S$	$[M-H]^-$			+++++
890.6405	890.6397	-0.0008	**SHexCer 42:1;O2**	$C_{48}H_{93}NO_{11}S$	$[M-H]^-$			+++++
894.6238	894.6230	-0.0008	**PS 44:4**	$C_{50}H_{90}NO_{10}P$	$[M-H]^-$			+++
896.6394	896.6386	-0.0008	**PS 44:3**	$C_{50}H_{92}NO_{10}P$	$[M-H]^-$			++
904.6180	904.6189	+0.0009	**SHexCer 42:2;O3***	$C_{48}H_{91}NO_{12}S$	$[M-H]^-$			++++
906.6354	906.6346	-0.0008	**SHexCer 42:1;O3**	$C_{48}H_{93}NO_{12}S$	$[M-H]^-$			++++
909.5495	909.5499	+0.0004	**PI 40:6**	$C_{49}H_{83}O_{13}P$	$[M-H]^-$			+++
913.5805	913.5812	+0.0007	**PI 40:4**	$C_{49}H_{87}O_{13}P$	$[M-H]^-$			++
1032.3688	1032.3689	+0.0001	**CoA 18:0***	$C_{39}H_{70}N_7O_{17}P_3S$	$[M-H]^-$			+++
1060.3997	1060.4002	+0.0005	**CoA 20:0**	$C_{41}H_{74}N_7O_{17}P_3S$	$[M-H]^-$			++

Table S3: List of identified lipids with high-intensity signals from the MSI experiment using the BB7 matrix in negative mode. The number of + symbols in the Intensity column indicates the order of magnitude of the signal.

Measured	Matched Mass	Delta	Lipid	Formula	Ion	Isobaric lipid	Ion	Intensity
301.2176	301.2173	-0.0003	**FA 20:5**	$C_{20}H_{30}O_2$	$[M-H]^-$	*ST 20:2;O2*	$[M-H]^-$	Covred with matix
303.2335	303.2330	-0.0005	**FA 20:4**	$C_{20}H_{32}O_2$	$[M-H]^-$	*ST 20:1;O2*	$[M-H]^-$	++++++
327.2328	327.2330	+0.0002	**FA 22:6**	$C_{22}H_{32}O_2$	$[M-H]^-$			++++++
329.2485	329.2486	+0.0001	**FA 22:5**	$C_{22}H_{34}O_2$	$[M-H]^-$	*SFE 22:5*	$[M-H]^-$	+++++
331.2634	331.2643	+0.0009	**FA 22:4**	$C_{22}H_{36}O_2$	$[M-H]^-$	*SFE 22:4*	$[M-H]^-$	+++++
333.2790	333.2799	+0.0009	**FA 22:3**	$C_{22}H_{38}O_2$	$[M-H]^-$	*SFE 22:3, WE 22:3*	$[M-H]^-$	++++
335.2949	335.2956	+0.0007	**FA 22:2**	$C_{22}H_{40}O_2$	$[M-H]^-$	*SFE 22:2, WE 22:2*	$[M-H]^-$	+++
337.3106	337.3112	+0.0006	**FA 22:1**	$C_{22}H_{42}O_2$	$[M-H]^-$	*SFE 22:1, WE 22:1*	$[M-H]^-$	++++
339.3260	339.3269	+0.0009	**FA 22:0**	$C_{22}H_{44}O_2$	$[M-H]^-$	*SFE 22:0, WE 22:0*	$[M-H]^-$	++++
-	385.2384	-	**ST 24:4;O4**	$C_{24}H_{34}O_4$	$[M-H]^-$			
391.2249	391.2255	+0.0006	**CPA 16:0***	$C_{19}H_{37}O_6P$	$[M-H]^-$			+++++
393.2411	393.2412	+0.0001	**LPA O-16:1**	$C_{19}H_{39}O_6P$	$[M-H]^-$			++++
409.2364	409.2361	-0.0003	**LPA 16:0**	$C_{19}H_{39}O_7P$	$[M-H]^-$			+++++
417.2416	417.2412	-0.0004	**CPA 18:1**	$C_{21}H_{39}O_6P$	$[M-H]^-$			+++++
419.2568	419.2568	0.0000	**CPA 18:0**	$C_{21}H_{41}O_6P$	$[M-H]^-$			+++++
435.2523	435.2517	-0.0006	**LPA 18:1***	$C_{21}H_{41}O_7P$	$[M-H]^-$			+++++
437.2681	437.2674	-0.0007	**LPA 18:0**	$C_{21}H_{43}O_7P$	$[M-H]^-$			+++++
463.2836	463.2830	-0.0006	**LPA 20:1**	$C_{23}H_{44}O_7P$	$[M-H]^-$			++++
480.3098	480.3096	-0.0002	**LPE 18:0***	$C_{23}H_{48}NO_7P$	$[M-H]^-$	*LPE O-18:1;O*<br>*LPC O-16:0*	$[M-H]^-$<br>$[M-CH_3]^-$	+++++
571.2880	571.2889	+0.0009	**LPI 16:0***	$C_{25}H_{49}O_{12}P$	$[M-H]^-$			++++
599.3192	599.3202	+0.0010	**LPI 18:0**	$C_{27}H_{53}O_{12}P$	$[M-H]^-$			+++++
673.4809	673.4814	+0.0005	**PA 34:1***	$C_{37}H_{70}O_8P$	$[M-H]^-$			+++++
699.4969	699.4970	+0.0001	**PA 36:2***	$C_{39}H_{73}O_8P$	$[M-H]^-$			+++++
701.5127	701.5127	0.0000	**PA 36:1**	$C_{39}H_{75}O_8P$	$[M-H]^-$			+++++
716.5232	716.5236	+0.0004	**PE 34:1***	$C_{39}H_{76}NO_8P$	$[M-H]^-$	*PC 32:1*	$[M-CH_3]^-$	++++
718.5392	718.5392	0.0000	**PE 34:0**	$C_{39}H_{78}NO_8P$	$[M-H]^-$	*PC 32:0*	$[M-CH_3]^-$	+++++
721.4808	721.4814	+0.0006	**PA 38:5***	$C_{41}H_{71}O_8P$	$[M-H]^-$			++++
723.4972	723.4970	-0.0002	**PA 38:4**	$C_{41}H_{73}O_8P$	$[M-H]^-$			+++++
726.5442	726.5443	+0.0001	**PE O-36:3**	$C_{41}H_{78}NO_7P$	$[M-H]^-$	*PC O-34:3*	$[M-CH_3]^-$	+++++
728.5600	728.5600	0.0000	**PE O-36:2**	$C_{41}H_{80}NO_7P$	$[M-H]^-$	*PC O-34:2*	$[M-CH_3]^-$	+++++
731.5020	731.5021	+0.0001	**PA O-40:7**	$C_{43}H_{73}O_7P$	$[M-H]^-$			++++
744.5546	744.5549	+0.0003	**PE 36:1***	$C_{41}H_{80}O_8P$	$[M-H]^-$	*PC 34:1*	$[M-CH_3]^-$	+++++
747.4977	747.4970	-0.0007	**PA 40:6**	$C_{43}H_{73}O_8P$	$[M-H]^-$			+++++
750.5446	750.5443	-0.0003	**PE O-38:5***	$C_{43}H_{78}O_7P$	$[M-H]^-$	*PC O-36:5*	$[M-CH_3]^-$	+++++
762.5080	762.5079	-0.0001	**PE 38:6**	$C_{43}H_{74}O_8P$	$[M-H]^-$	*PC 36:6*	$[M-CH_3]^-$	+++++

766.5391	766.5392	+0.0001	**PE 38:4**	$C_{43}H_{78}NO_8P$	$[M-H]^-$			+++++
772.5289	772.5287	-0.0002	**PE O-40:8**	$C_{45}H_{76}NO_7P$	$[M-H]^-$			++++
774.5440	774.5443	+0.0003	**PE P-40:6**	$C_{45}H_{78}NO_7P$	$[M-H]^-$			+++++
778.5754	778.5756	+0.0002	**PE O-40:5**	$C_{45}H_{82}NO_7P$	$[M-H]^-$	*PC O-38:5*	$[M-CH_3]^-$	+++++
788.5233	788.5236	+0.0003	**PE 40:7**	$C_{45}H_{76}NO_8P$	$[M-H]^-$	*PC 38:7*	$[M-CH_3]^-$	++++
790.5386	790.5392	+0.0006	**PE 40:6**	$C_{45}H_{78}NO_8P$	$[M-H]^-$	*PE O-40:7;O*	$[M-H]^-$	+++++
794.5702	794.5705	+0.0003	**PE 40:4**	$C_{45}H_{82}NO_8P$	$[M-H]^-$	*PC 38:4*	$[M-CH_3]^-$	+++++
806.5463	806.5458	-0.0005	**SHexCer 36:1;O2***	$C_{42}H_{81}NO_{11}S$	$[M-H]^-$			++++
816.5541	816.5549	+0.0008	**PE 42:7**	$C_{47}H_{80}NO_8S$	$[M-H]^-$	*PC 40:7*	$[M-CH_3]^-$	+++
822.5404	822.5407	+0.0003	**SHexCer 36:1;O3**	$C_{42}H_{81}NO_{12}S$	$[M-H]^-$			++++
824.5659	824.5658	-0.0001	**IPC 36:0;O3**	$C_{42}H_{84}NO_{12}P$	$[M-H]^-$			+++
834.5291	834.5291	0.0000	**PS 40:6**	$C_{46}H_{78}NO_{10}P$	$[M-H]^-$			+++++
834.5774	834.5771	+0.0003	**SHexCer 38:1;O2**	$C_{44}H_{85}NO_{11}S$	$[M-H]^-$			++++
838.5598	838.5604	+0.0006	**PS 40:4**	$C_{46}H_{82}NO_{10}P$	$[M-H]^-$			++++
850.5728	850.5720	-0.0008	**SHexCer 38:1;O3***	$C_{44}H_{85}NO_{12}S$	$[M-H]^-$			++++
857.5181	857.5186	+0.0005	**PI 36:4**	$C_{45}H_{79}O_{13}P$	$[M-H]^-$			+++++
862.6088	862.6084	-0.0004	**SHexCer 40:1;O2**	$C_{46}H_{89}NO_{11}S$	$[M-H]^-$			++++
878.6040	878.6033	-0.0007	**SHexCer 40:1;O3**	$C_{46}H_{89}NO_{12}S$	$[M-H]^-$			+++++
883.5351	883.5342	-0.0009	**PI 38:5**	$C_{47}H_{81}O_{13}P$	$[M-H]^-$			++++
885.5499	885.5499	0.0000	**PI 38:4**	$C_{47}H_{83}O_{13}P$	$[M-H]^-$			+++++
888.6237	888.6240	+0.0003	**SHexCer 42:2;O2**	$C_{48}H_{91}NO_{11}S$	$[M-H]^-$			+++++
890.6388	890.6397	+0.0009	**SHexCer 42:1;O2**	$C_{48}H_{93}NO_{11}S$	$[M-H]^-$			+++++
894.6221	894.6230	+0.0009	**PS 44:4**	$C_{50}H_{90}NO_{10}P$	$[M-H]^-$			+++
896.6381	896.6386	+0.0005	**PS 44:3**	$C_{50}H_{92}NO_{10}P$	$[M-H]^-$			+++
904.6189	904.6189	0.0000	**SHexCer 42:2;O3***	$C_{48}H_{91}NO_{12}S$	[M - H]-			+++++
906.6348	906.6346	-0.0002	**SHexCer 42:1;O3**	$C_{48}H_{93}NO_{12}S$	[M - H]-			+++++
909.5497	909.5499	+0.0002	**PI 40:6**	$C_{49}H_{83}O_{13}P$	[M - H]-			++++
913.5805	913.5812	+0.0007	**PI 40:4**	$C_{49}H_{87}O_{13}P$	[M - H]-			+++
-	1032.3689	-	**CoA 18:0***	$C_{39}H_{70}N_7O_{17}P_3S$	[M - H]-			
-	1060.4002	-	**CoA 20:0**	$C_{41}H_{74}N_7O_{17}P_3S$	[M - H]-			

Table S4: List of identified lipids with high-intensity signals from the MSI experiment in using the DAN matrix negative mode. The number of + symbols in the Intensity column indicates the order of magnitude of the signal.
